# Calcium phosphate engineered photosynthetic microalgae to combat hypoxic-tumor by *in-situ* modulating hypoxia and cascade radio-phototherapy

**DOI:** 10.7150/thno.55441

**Published:** 2021-01-22

**Authors:** Danni Zhong, Wanlin Li, Shiyuan Hua, Yuchen Qi, Tingting Xie, Yue Qiao, Min Zhou

**Affiliations:** 1 Department of Surgery, The Fourth Affiliated Hospital, Zhejiang University School of Medicine, Yiwu 322000, China.; 2 Institute of Translational Medicine, Zhejiang University, Hangzhou 310009, China.; 3 State Key Laboratory of Modern Optical Instrumentations, Zhejiang University, Hangzhou, 310058, China.

**Keywords:** Chlorella vulgaris, chlorophyll, photosynthetic microalgae, tumor hypoxia, radiotherapy, phototherapy

## Abstract

**Rationale:** Hypoxia is one of the crucial restrictions in cancer radiotherapy (RT), which leads to the hypoxia-associated radioresistance of tumor cells and may result in the sharp decline in therapeutic efficacy.

**Methods:** Herein, living photosynthetic microalgae (Chlorella vulgaris, *C. vulgaris*), were used as oxygenators, for *in situ* oxygen generation to relieve tumor hypoxia. We engineered the surface of *C. vulgaris* (CV) cells with calcium phosphate (CaP) shell by biomineralization, to form a biomimetic system (CV@CaP) for efficient tumor delivery and in-situ active photosynthetic oxygenation reaction in tumor.

**Results:** After intravenous injection into tumor-bearing mice, CV@CaP could remarkably alleviate tumor hypoxia by continuous oxygen generation, thereby achieving enhanced radiotherapeutic effect. Furthermore, a cascade phototherapy could be fulfilled by the chlorophyll released from photosynthetic microalgae combined thermal effects under 650 nm laser irradiation. The feasibility of CV@CaP-mediated combinational treatment was finally validated in an orthotropic breast cancer mouse model, revealing its prominent anti-tumor and anti-metastasis efficacy in hypoxic-tumor management. More importantly, the engineered photosynthetic microalgae exhibited excellent fluorescence and photoacoustic imaging properties, allowing the self-monitoring of tumor therapy and tumor microenvironment.

**Conclusions:** Our studies of this photosynthetic microsystem open up a new dimension for solving the radioresistance issue of hypoxic tumors.

## Introduction

Radiotherapy (RT) has been widely used for the clinical treatment of cancer, using high-intensity ionizing radiation beams to destroy tumors without depth restriction, during which it can produce cytotoxic reactive oxygen species (ROS) to induce DNA double-strand damage 1-4. During RT, oxygen plays a key role in sensitizing radiation-induced cell killing by promoting the production of excess ROS and preventing the self-repair of DNA damage 5-7. Unfortunately, the hypoxia condition that exists in primary solid tumors leads to the radioresistance of tumor cells, greatly reducing the therapeutic efficiency and further increasing the risk of damage to normal tissue caused by the high dosage of irradiation 8-11. Therefore, developing effective approaches to relieve tumor hypoxia, increase the radiosensitivity of hypoxic cells and substantially enhance the RT efficacy would be clinically valuable for cancer treatment.

In recent years, nanoparticle-based radiosensitizers have been heavily studied in tumor hypoxia regulation for enhanced RT, such as hemoglobin- and perfluorocarbon-loaded nanoparticles 12-14 for tumor targeted oxygen-carrying, or metal-organic frameworks (MOFs) 15,16, quantum dots (QDs) 17,18, and metal-based nanoparticles 19-21, for intratumoral oxygen generation. However, the field itself is still in its infancy with many problems. For example, the feasibility of these oxygen supply systems for large-scale applications is limited by several restrictions, including complicated chemical synthesis, high production costs, serious technical challenges and so on 22-24. Besides, whether the nanomedicine is effective for radiosensitization *in vivo* depends greatly on the tumor microenvironment (TME), because it mainly works by triggering the decomposition of H_2_O_2_ existing in the TME into water and oxygen to ameliorate tumor hypoxia 25-27. More importantly, a number of investigations have revealed that many nanomaterials exhibit minor to major nanotoxicity to biological systems via broad underlying mechanisms, including physical, chemical, or immunological 28, 29. In terms of the chemical dimension alone, their composition, surface chemistry, as well as the released ions, as foreign materials, may trigger serve systemic toxicity 30, 31. Considering the above limiting factors, it is highly desired to design an advanced, facile, and practical strategy to modulate the hypoxia in solid tumors, so as to achieve optimal therapeutic outcomes of RT.

**Scheme 1:**
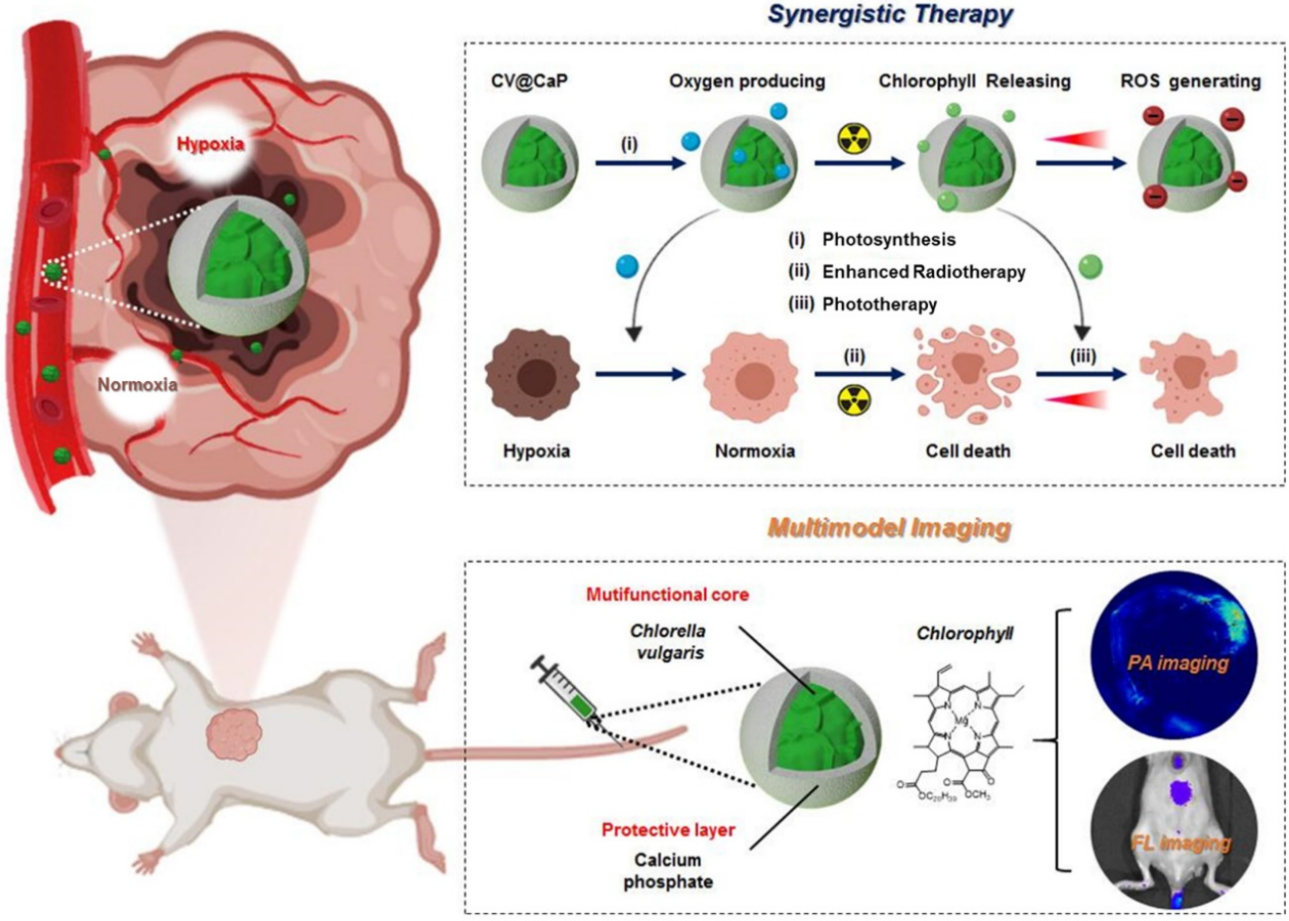
**Schematic illustration of biomineralized *C. vulgaris* mediated PAI/FLI guided synergistic therapy.** Mechanistic representative diagram of the self-enhanced radio-phototherapy: (i) CV@CaP as a photosynthetic system produced large amounts of oxygen *in situ* to modulate tumor hypoxia. (ii) The alleviation of tumor hypoxia further improved the RT efficacy. (iii) X-ray induced chlorophyll releasing as photosensitizer, generating ROS under 650 nm laser, in combination with thermal effects to realize phototherapy.

Herein, we developed an engineered natural biosystem, *Chlorella vulgaris* (*C. vulgaris*), a photosynthetic microalgae, coated with calcium phosphate (CaP) protective layer to modulate tumor hypoxia for dual-modal imaging-guided synergistic therapy (**Scheme 1**). In this CaP engineered microalgae system (CV@CaP), the CaP shield allowed the microalgae cells to pass through a hostile milieu to reach the tumor sites while maintaining their photosynthetic activity. The CV@CaP capable of photosynthesis alleviated the tumor hypoxia by *in situ* oxygen producing, and significantly enhanced the RT effect. At the same time, the chlorophyll released from CV@CaP generated large amounts of ROS upon 650 nm laser irradiation, achieving both photodynamic therapy (PDT) and laser-induced photothermal therapy (PTT). Most importantly, the high concentration of chlorophyll in the *C. vulgaris* cells could be used as a highly versatile tool for fluorescent imaging (FLI) and photoacoustic imaging (PAI) to guide tumor treatment. Overall, the usage of natural microalgae, as oxygenerators, is expected to lay the groundwork for the next generation of biocompatible, multifunctional and novel radiosensitizers to overcome the hypoxia-related RT resistance.

## Methods

### Materials

Calcium chloride (97%, CaCl_2_•6H_2_O), Sodium phosphate dibasic (99%, Na_2_HPO_4_), were obtained from MACKLIN (Shanghai, China). *Chlorella vulgaris* (CV) and BG11 medium were purchased from Guangyu Biological Technology (Shanghai, China). Trypsin-EDTA, Dulbecco's modified eagle's medium (DMEM), fetal bovine serum (FBS) were obtained from Gibco-BRL (Burlington, ON, Canada). All chemicals and reagents were used without further purification.

### Characterization

The bright-field and fluorescence images of original chlorella cells were captured by an optical microscope (Zeiss, Germany). The size, morphology and EDS spectra of CV@CaP were acquired using a field emission scanning electron microscopy (FESEM, Hitachi SU-70, Japan) operated at 3 kV and high-resolution transmission electron microscopy (HRTEM, Tecnai F20, FEI, USA) operated at 200 kV. The FTIR patterns were characterized by a Nicolet Nexus 470 (Madison, USA). The dynamic light-scattering diameter and zeta potential were measured using a Zetasizer Nano-ZS90 instrument (Malvern, UK). The UV-Vis spectra were recorded with a UV-2600 spectrophotometer (Shimadzu, Kyoto, Japan). The fluorescence spectra were recorded using an RF-6000 fluorescence spectrophotometer (Shimadzu, Kyoto, Japan) with an excitation wavelength at 475 nm.

### Synthesis of CV@CaP

*Chlorella* cells were grown in BG11 medium (0.1% ammonium ferric citrate green, 0.1% citric acid, 0.1% CaCl_2_•2H_2_O, 0.1% EDTANa_2_, 0.1% K_2_HPO_4_, 0.1% MgSO_4_•7H_2_O, Na_2_CO_3_, 0.2% NaCl and 0.1% tracemetal sol) incubated at 25°C with shaking at 250 rpm. CV@CaP were synthesized based on a biomineralization process 37, 46: *Chlorella* cells were collected by centrifugation (3000 rpm, 10 min) and washed with PBS. To prepare the shell of calcium phosphate, 10 mL of a mixture, which contained the chlorella (1 × 10^6^ cells/mL) and 2 mM CaCl_2_, was stirred and titrated with 10 mL Na_2_HPO_4_ (2 mM) at a rate of 1 mL/min. The obtained CV@CaP cells were washed with PBS three times and stored in a BG11 medium at 4 °C.

### Cell viability

Murine breast cancer cells (4T1) were cultured in DMEM containing 4500 mg/L glucose, 10% FBS and 1% antibiotics (100 μg/mL streptomycin and 100 U/mL penicillin) at 37 °C in a 5% CO_2_ atmosphere. For evaluation of the cytotoxicity of chlorella before and after surface modification, cells were seeded in a 96-well plate at a density of 5 × 10^3^ cells per well and incubated for 24 h. Then, the medium of the cells was replaced by varying concentrations of chlorella and CV@CaP (0, 4, 20, 100, 500, 2500 × 10^4^/mL, n = 5), followed by co-culture for another 24 h. Cell viability was evaluated using the standard thiazolyltetrazolium (MTT) assay kit (YEASEN, Shanghai, China), and quantified by measuring the absorbance at 490 nm with a SpectraMax M5 plate reader (Molecular Devices, USA).

### Oxygen production performance

The oxygen production performance of *chlorella* before and after coating was evaluated using an oxygen-sensing electrode (Unisense, DK). Firstly, 50 mL of *chlorella* and CV@CaP (containing 5 × 10^7^ cells) samples were cultured in Erlenmeyer flasks overnight in darkness to remove the dissolved oxygen. A standard curve was established through sensing a zero-oxygen solution containing 0.1 M ascorbic acid and 0.2 M sodium hydroxide and an oxygen saturated solution. The corresponding oxygen production efficiency of each sample was detected successively for 5 h under exposure to a bright light according to the obtained standard curve. To evaluate the oxygen supplementation of CV@CaP in hypoxic tumor cells, 1 × 10^5^ 4T1 cells were seeded in a 6-well plate and incubated overnight at 37 °C in a 21% O_2_ (Normoxia) or 1% O_2_ (Hypoxia) air humidified atmosphere, respectively. The hypoxic cells were then treated with or without CV@CaP (1 × 10^6^ cells/mL) for 4 h under a bright light. After different treatments, cells were stained with DAPI and [Ru(dpp)_3_]Cl_2_ for visualizing the nuclei and hypoxia under a fluorescence microscope.

### Colony formation assay and cell migration assay

Hypoxic cells were obtained by incubating 4T1 cells at 37 °C in 1% O_2_ atmosphere. 1 × 10^5^ hypoxic cells were incubated with or without 2 mL of CV@CaP (1 × 10^6^ cells/mL) in a 6-well plate for 4 h under white light, followed by exposure to X-ray at doses of 0, 2, 4, 6, and 8 Gy. 2 × 10^3^ cells were taken from each group (n = 3) and cultured under normal conditions for another 5 days. The colony formation was observed by staining with Giemsa (YEASEN, Shanghai, China) and quantified by counting the cell colonies (≥ 50 cells). For cell migration assay, upon reaching about 95% confluence, hypoxic cells were co-cultured with CV@CaP (1 × 10^6^ cells/mL) for 4 h under white light and treated with X-rays (0 and 6 Gy, n = 3). The cell layer was scraped off with a sterile 0.1 mL pipette tip to form a gap, and cell migration was calculated by the width of the gap at selected time points.

### DCFH-DA staining and live/dead cell staining

ROS generation was evaluated using a DCFH-DA assay kit (YEASEN, Shanghai, China). 4T1 cells were seeded in 96-well plates at a density of 1 × 10^4^ cells per well and incubated overnight. Cells were then incubated with pre-irradiated CV@CaP (1 × 10^6^ cells/mL) for 4 h, and exposed to a 650 nm laser at 1.0 W/cm^2^ for 3 min, before DCFH-DA assay. The generated ROS in cells was imaged under a fluorescence microscope and further quantified by a flow cytometer (Beckman, California, USA). For Live/dead cells staining, after being incubated with or without CV@CaP (1 × 10^6^ cells/mL) for 4 h under white light, cells were exposed to X-ray irradiation (6 Gy) and followed by 650 nm laser (1.0 W/cm, 3 min). Treated cells were stained using a Calcein-AM/PI double stain kit (YEASEN, Shanghai, China) and imaged under the fluorescence microscope.

### *In vivo* fluorescence imaging and photoacoustic imaging

Female Balb/c mice (6 weeks) were inoculated with 1 × 10^6^ 4T1 cells into the breast pad of the mice to establish the orthotopic tumor model. When the volume of orthotopic 4T1 tumors reached about 250 mm^3^, mice were intravenously (i.v.) injected with 200 μL of CV@CaP (1 × 10^7^ cells/mL) and photographed using a small animal live imaging system (IVIS Lumina LT Series III, Perkin Elmer, USA) before and after intravenous injection at 10 min, 30 min, 2 h, 4 h, 6 h, 8 h, 12 h, and 24 h, respectively. At animal euthanization, mice were sacrificed and tumor, brain, heart, liver, spleen, lung, kidney, stomach, small intestine, large intestine, bladder, muscle, bone, and marrow were collected and photographed as well. The total counts of each organ were quantified by Living Image 4.5 software (Perkin Elmer, USA). Photoacoustic images were acquired at different time points (0, 1, 2, 4 and 6 h) pre- and post-injection by a real-time multispectral optoacoustic tomographic imaging system (MSOT, inSight/inVision 25, iThera Medical, Germany) at the wavelength of 680 nm.

### Pharmacokinetics and metabolic analysis

The calibrated fluorescence standard curve was obtained by measuring the fluorescence intensity of samples with different amounts of CV@CaP dissolved in ethanol. Female Balb/c mice (6 weeks, n = 3) were i.v. injected with 200 μL of CV@CaP (1 × 10^7^ cells/mL), and blood from their retinal vein was collected before and after intravenous injection at 5 min, 10 min, 20 min, 40 min, 1 h, 2 h, 4 h, 8 h, 12 h, and 24 h, respectively. The blood samples then were clotted at room temperature for 30 minutes, and serum obtained by centrifugation (4000 rpm, 10 min). The serum samples were then diluted with ethanol and the fluorescence intensity of CV@CaP in the serum was measured by RF-6000 fluorescence spectrophotometer. For the metabolic analysis, female Balb/c mice (6 weeks, n = 3) were i.v. injected with 200 μL of CV@CaP (1 × 10^7^ cells/mL). At each predetermined time point, urine and faecal samples of mice were collected into 1.5 mL centrifuge tube, and photographed using small animal live imaging system. The urine samples were diluted with PBS.

### *In vivo* synergistic anticancer effects

Female Balb/c mice (6 weeks) were inoculated with 1 × 10^6^ 4T1 cells into the breast pad of the mice to establish the orthotopic tumor model. When the volume of orthotopic 4T1 tumors reached about 40 mm^3^, tumor-bearing mice were randomly divided into eight groups (n = 5, each group): 1) Control received PBS i.v. injection only; 2) CV@CaP; 3) Laser; 4) RT; 5) RT + Laser; 6) CV@CaP + Laser; 7) CV@CaP + RT and 8) CV@CaP + RT + Laser. All groups were i.v. injected with 200 μL of PBS or CV@CaP (1 × 10^7^ cells/mL) before treatment. Laser groups received 650 nm laser (1 W/cm^2^, 3 min), and RT groups received X-ray (6 Gy). Mice were exposed to a bright condition for 4 h after the administration, and then received radiotherapy, followed by phototherapy. The tumor volume and the body weight of mice were monitored every two days during the whole treatment (18 days). The tumor volume V (mm^3^) was calculated according to the following formula: V = length × width^2^/2. At the 18 d, all mice were photographed by a digital camera (SONY, Japan). Tumors and major organs from each group of mice were dissected, photographed, weighed, and processed for histological evaluation. Tumor tissues were then sectioned and stained for CD31, Ki-67, and HIF-1α staining. All sections were visualized using a virtual microscopy system (OlympusVS120, Tokyo, Japan).

### Statistical analysis

All statistical analyses were performed using the SPSS software package (SPSS, Inc., USA). All error bars used in this study are mean ± s.d. of at the least three independent experiments. All the data in this study used the two-tailed Student's t-test to analyze statistical significance. Statistically significant p values are indicated in Figures and/or legends as ***, p <0.001; **, p <0.01; *, p <0.05.

## Results and Discussion

### Fabrication of CV@CaP

**Figure 1:**
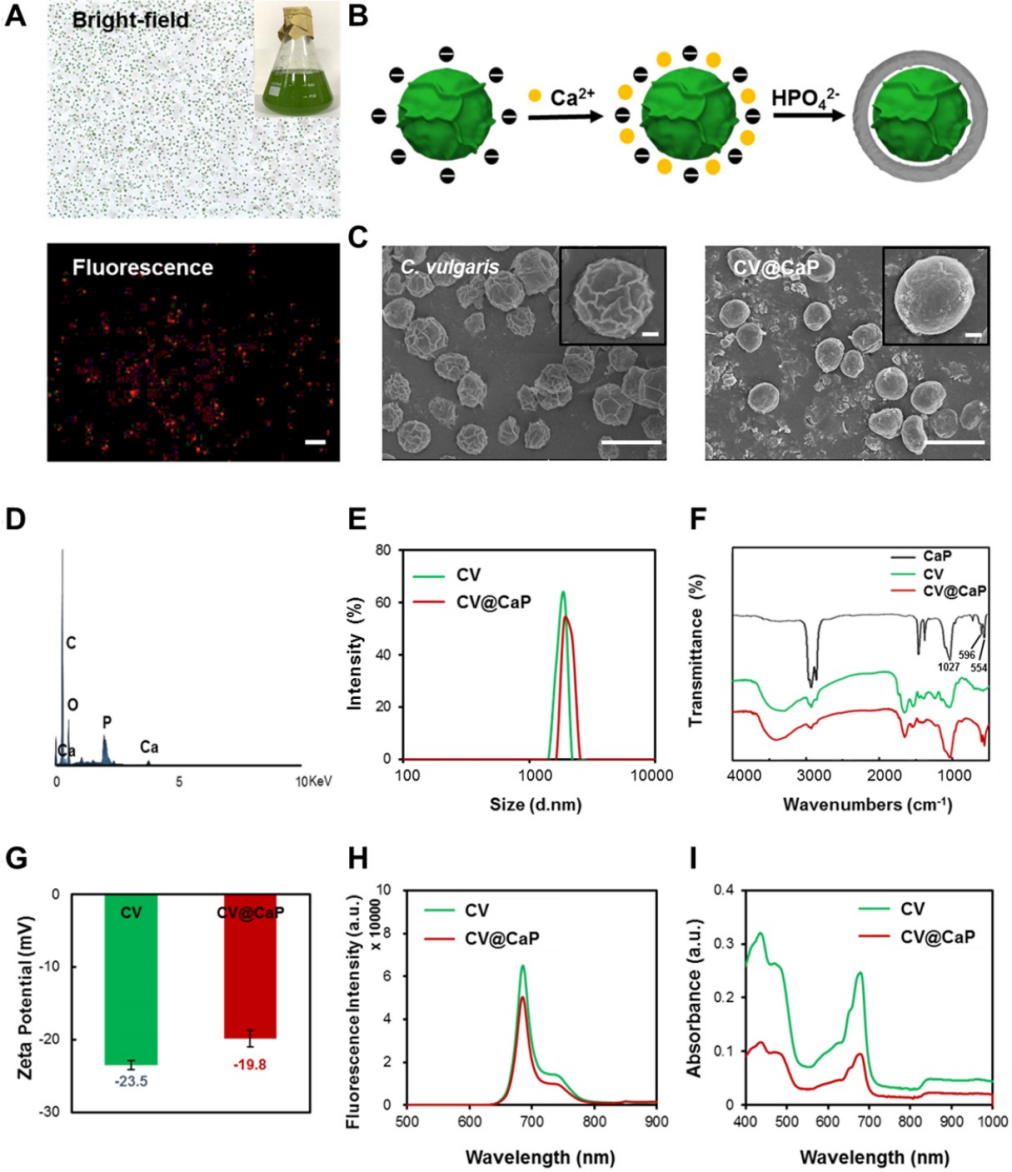
**Biomineralization of *C. vulgaris*.** (A) Bright (top) and fluorescence images (bottom) of native *C. vulgaris* cells (inset: a large-scale culture of CV). (B) Schematic illustration of the CaP mineralization process of *C. vulgaris*, experiencing the precipitation of calcium phosphate. (C) SEM images of the bare *C. vulgaris* cells (left) and the mineralized CV@CaP cells (right), scale bar = 5 µm, top insert: enlarged images of the cell, scale bar = 0.5 µm. (D) EDS analysis of CV@CaP, evidencing the presence of Ca and P. (E) DLS of CV and CV@CaP. (F) FTIR patterns of CaP, CV and CV@CaP. (G) Zeta potential, (H) fluorescence and (I) UV-Vis spectra of CV and CV@CaP.

*C. vulgaris* (CV), a microorganism abundant in nature, possess uniform spherical appearance and innate chlorophyll with fluorescence characteristics (**Fig. 1A**). Some kinds of unicellular organisms can use the outer membrane as a template for biomineralization, forming a functional protective coating 32, 33. Unlike them, *C. vulgaris* cannot induce mineralization spontaneously on its surface. Therefore, surface engineering of *C. vulgaris* is highly necessary to ensure its application *in vivo*. Herein, we chose biomineralization to modify *C. vulgaris*, instead of the previously reported erythrocyte membrane coating method 34, because the raw materials and reagents used in biomineralization are cheap and easy to obtain, and the reaction time is relatively short, laying a solid foundation for its future industrialization. Most importantly, bioinorganic materials are advantageous for surface engineering due to their good biocompatibility, biodegradability and nontoxicity 35. Among them, calcium phosphate (CaP), as a non-immunogenic and environmentally friendly biomaterial, has been used to engineer biological substrates including proteins, bacteria and viruses in recent years to ensure their stability and environmental resistance 36, 37. To fabricate the CaP shell, we used a facile dip-coating method as **Fig. 1B** illustrated. The positively charged calcium ions were firstly bonded to the negatively charged surface of *C. vulgaris* cells, and then *in situ* mineralization after the supplementary phosphate ions. The precipitation of calcium phosphate provided a mineral covering to protect the inner photosynthetic microalgae cells. Scanning electron microscopy (SEM) images (**Fig. 1C**) showed that the surface of native *C. vulgaris* typically was rough and textured, which became smooth and dense after mineral coating. Compare to original *C. vulgaris*, calcium phosphate-functionalized *C. vulgaris* (CV@CaP) has a uniform layer according to transmission electron microscopy (TEM) images (**Fig. S1**). EDS analysis (**Fig. 1D**) further confirmed the presence of calcium and phosphorus in the outer layer. CV@CaP were approximately 2600 nm, larger than the uncloaked CV with an average diameter of 2400 nm (**Fig. 1E**), evidencing the existence of the covering layer. Additionally, the infrared absorption peaks of the phosphate group (554 cm^-1^, 596 cm^-1^, and 1027 cm^-1^) were observed in CV@CaP sample compared with CV due to the deposition of minerals (**Fig. 1F**). Besides, the negative charge on the surface of CV also is shielded by the resultant thin mineral layer (**Fig. 1G**). Interestingly, the intrinsic features and fluorescent properties of photosynthetic microalgae core could be well preserved after biomineralization, attributing to the noninvasive coating process (**Fig. 1H, I**). Compared with naked *C. vulgaris*, CV@CaP was more resistant to degradation in the physiological environment (**Fig. S2**), suggesting that the mineralized shell could help the protection and delivery of microalgae cells* in vivo*.

### Oxygen generation of CV@CaP

**Figure 2:**
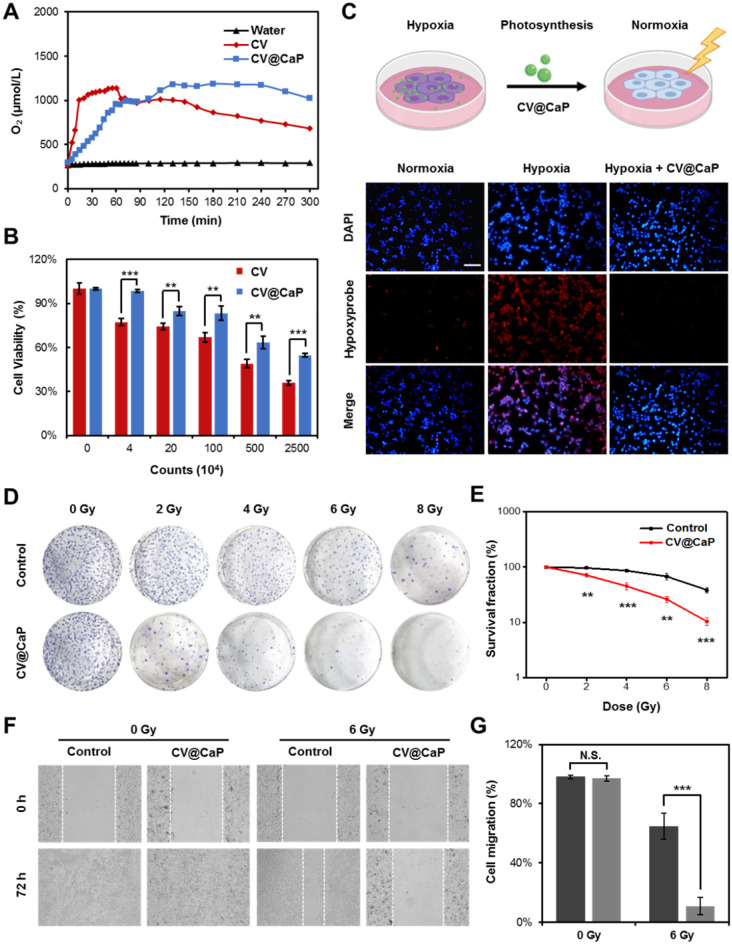
**Oxygeneration performance of CV@CaP to enhance RT efficiency* in vitro*.** (A) Oxygen production curves of water, CV and CV@CaP over time (5 hours). (B) Cytotoxicity of CV and CV@CaP to breast cancer cell line 4T1. (C) The process (top) of CV@CaP modulating cell hypoxia by photosynthesis, and fluorescence images (bottom) showing the alleviation of hypoxia in the 4T1 tumor cells treated with CV@CaP. Cells and hypoxia signals were stained with DAPI (blue) and hypoxyprobe (red), respectively. Scale Bar = 100 µm. (D) Colony formation assay and (E) Survival curves of 4T1 cells treated with or without CV@CaP under X-ray irradiation (0 to 8 Gy). (F) Cell migration assay and (G) corresponding quantitative analysis of 4T1 cells treated with or without CV@CaP under X-ray irradiation (0 and 6 Gy). **p < 0.01, ***p < 0.001.

To study the effect of artificial shells on the biological functionalities of *C. vulgaris* cells, we first investigated the oxygen generation capacity we most interested in. As shown in **Fig. 2A**, we observed that *C. vulgaris* cells could produce large amounts of O_2_ within 60 min under a bright light (**Fig. S3**), attributed to the high efficiency of photosynthesis. Differently, the enclosed cells appeared inert after surface modification, in which O_2_ generation peaked 1 h later than the native *C. vulgaris* cells. Nevertheless, the oxygen supply showed a time-dependent increase and could prolong up to 4 h. This property ensured CV@CaP sufficient time to reach the tumor site and effectively modulate the hypoxic microenvironment by producing oxygen. Furthermore, the mineral shell isolated the cell from the external environment, effectively improving its biosecurity. Compared with the bare CV cells, CV@CaP exhibited lower cytotoxicity even at high concentrations (**Fig. 2B**). Based on the above results, we then evaluated the efficacy of CV@CaP in alleviating hypoxia to improve the radiation therapeutic effects at the *in vitro* level.

First, the oxygenation capacity of CV@CaP for modulating cells from hypoxic conditions (1% O_2_) was assessed. After co-culture with CV@CaP, the intracellular hypoxia signal (red fluorescence) level, indicated by [Ru(dpp)_3_]Cl_2_ probe, was obviously decreased in the hypoxic cells, which was basically consistent with normal cells (**Fig. 2C** and **Fig. S4**). During radiotherapy, oxygen was able to sensitize radiation-induced cell killing effect but the sensitization was restricted in hypoxic tumor cells, resulting in its resistance to irradiation. We then evaluated the enhanced RT effect after CV@CaP alleviating the hypoxia of cancer cells. As the colony formation (**Fig. 2D** and**Fig. S5**) showed, CV@CaP-treated hypoxic 4T1 cancer cells exhibited a remarkable radiosensitization effect, compared to X-ray alone. Corresponding survival fraction curve demonstrated the CV@CaP mediated-RT enhancement was dose-dependent from 0 to 8 Gy, leading over a 90% inhibition on cell proliferation at 8 Gy (**Fig. 2E**). Besides, the sensitizing efficacy was also found in the inhibition of metastasis and cell migration (**Fig. 2F**). Almost 65% of cell migration was achieved in the control group irradiated by X-ray with a dose of 6 Gy, but only 11% in the CV@CaP-treated group (**Fig. 2G**). All these above results preliminarily confirmed the potency of CV@CaP in relieving hypoxia to enhance the RT efficiency.

### CV@CaP-mediated *in vitro* phototherapeutic effect

**Figure 3:**
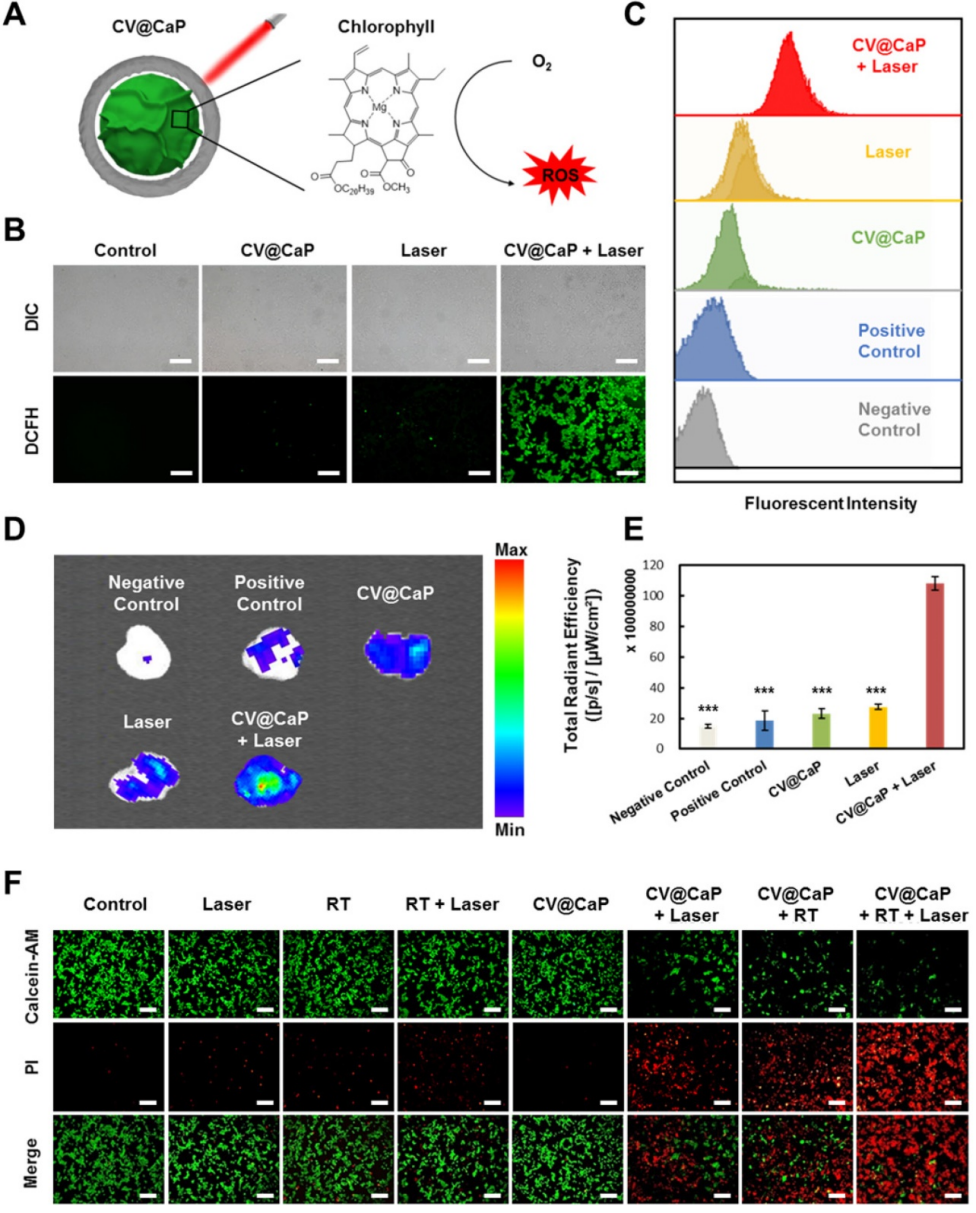
** Chlorophyll-induced ROS generation combined with thermal effects for phototherapy *in vitro*.** (A) Chlorophyll released from CV@CaP could be used as photosensitizer to generate ROS under 650 nm laser irradiation (1.0 W/cm, 3 min) for photodynamic therapy. (B) Fluorescence images of 4T1 cells stained with DCFH-DA after different treatments. CV@CaP were pre-treated with X-ray. Scale Bar = 200 µm. (C) Flow cytometry analysis showing the expression of ROS in 4T1 cells after various treatments. CV@CaP were pre-treated with X-ray. (D) IVIS images and (E) quantification of the total radiant efficiency of ROS signals in the dissected tumors after various treatments. (F) Fluorescence images of live/dead staining of 4T1 cells in different treated groups. The cells were co-stained with Calcein-AM (green, living cells) and PI (red, dead cells). Scale Bar = 100 µm. ***p < 0.001.

Furthermore, we studied the CV@CaP-mediated *in vitro* phototherapeutic effect, attributing to the release of chlorophyll, a common photosensitizer, from the *C. vulgaris* core (**Fig. 3A**), combined with the photothermal effects induced by laser. As shown in **Fig. S6**, large amounts of chlorophyll were detected after the X-ray treatment (6 Gy) due to the destruction of the *C. vulgaris* structure. To explore the possible mechanism of chlorophyll-induced PDT, DCFH-DA staining, a reactive oxygen species (ROS) indicator, was applied *in vitro* and *in vivo*. Under 650 nm laser irradiation, cells co-incubated with X-ray treated CV@CaP produced a much stronger ROS green fluorescence signal than other treatments (**Fig. 3B** and **Fig. S7**). A similar result was revealed in flow cytometry assay that X-ray treated CV@CaP plus laser irradiation-induced ROS in 93.2% of 4T1 cells, whereas no more than 46.7% in any other group (**Fig. 3C** and **Fig. S8**). However, cells co-incubated with untreated CV@CaP produced fewer ROS, confirming that X-ray contributed to the release of photosensitizer (**Fig. S9**). Subsequently, the CV@CaP-induced ROS effect was further investigated *in vivo* by DCFH-DA staining. The total fluorescence intensity of the intratumoral ROS significantly increased in the CV@CaP combined laser group, indicating the good performance of CV@CaP in leading a strong PDT efficacy (**Fig. 3D, E**). To assess the synergistic antitumor effect resulting from CV@CaP-based radiosensitization together with phototherapy, calcein-AM/propidium iodide (Calcein-AM/PI) staining was used *in vitro* (**Fig. 3F**). Treatment by CV@CaP assisted with X-ray or laser could cause a large number of dead cells (red fluorescence), compared to other control groups, which were mostly living cells (green fluorescence). These results demonstrated that CV@CaP could not only effectively increase intracellular oxygen, realizing radiosensitization, but also induce thermal effects under laser to kill tumor cells. Notably, CV@CaP mediated radio-phototherapy offered a significant cell killing efficacy (**Fig. S10**, P>0.001), suggesting the great potential for CV@CaP in combinatorial anticancer therapy.

### Fluorescence-based *in vivo* imaging and biodistribution

**Figure 4:**
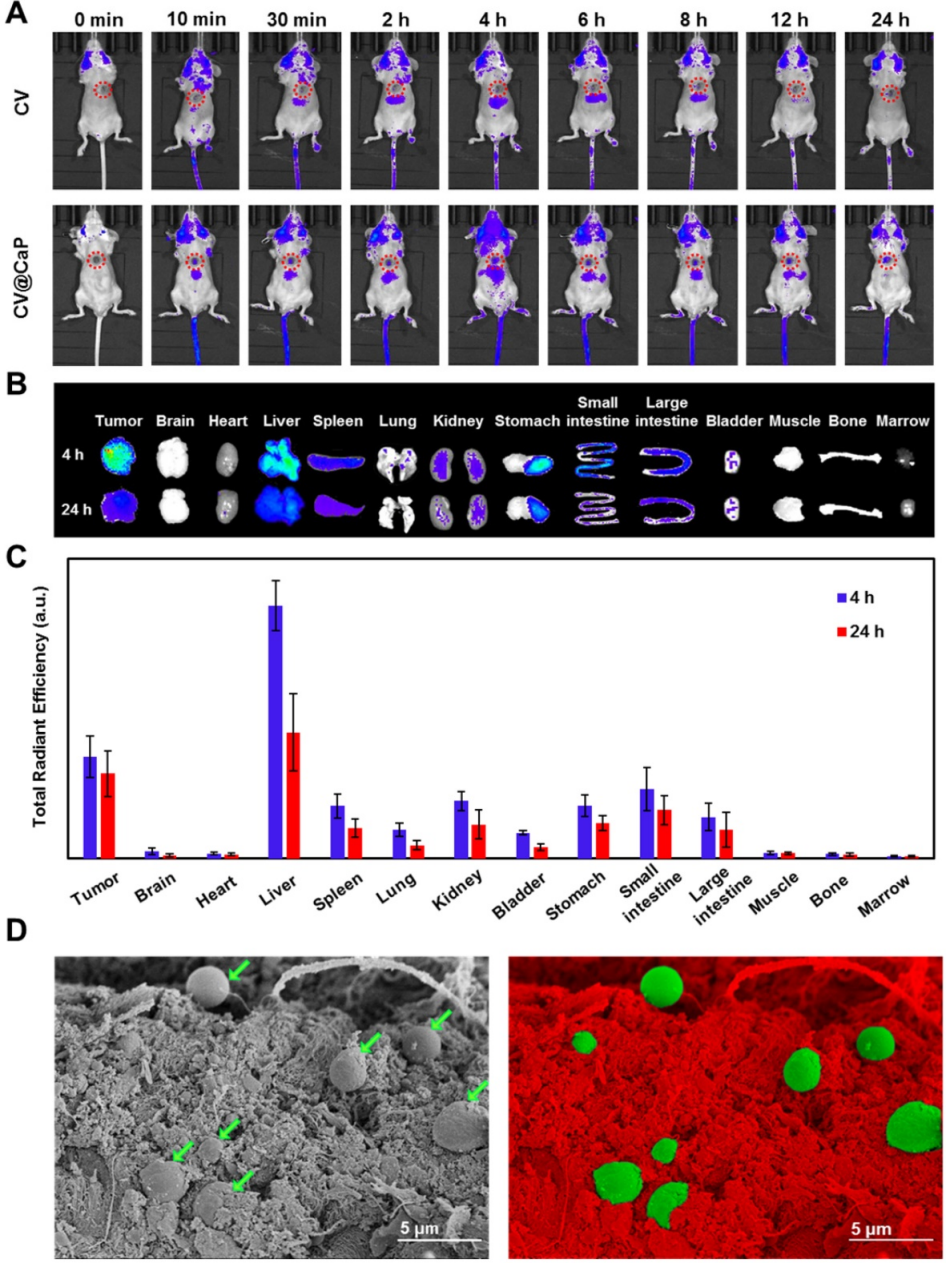
*** In vivo* fluorescence imaging and biodistribution of CV@CaP.** (A) Time-dependent *in vivo* FL images of 4T1 tumor-bearing mice (red circles: the tumor sites) after intravenous injection of CV and CV@CaP. (B) *Ex vivo* FL images of major organs and tissues from 4T1 tumor-bearing mice after 4 h and 24 h intravenous injection of CV@CaP. (C) Biodistribution of CV@CaP in major organs and tissues quantitated from *ex vivo* FLI analysis. (D) SEM (left) and pseudo-color (right) images showing the accumulation of CV@CaP (green) in the tumor tissues (red). Scale Bar = 5 µm.

The innate autofluorescence property of photosynthetic microalgae made it possible for the applications of fluorescence-based imaging. To verify that, the *in vivo* fluorescence imaging was assessed by orthotopic 4Τ1 tumor-bearing mice after i.v. injection with CV and CV@CaP. The fluorescence signals observed in the tumor site (red circles) of the mice injected with CV@CaP started immediately at 10 min post-injection, gradually enhanced, peaked at 4 h, and almost lasted up to 24 h (**Fig. 4A**). At 4 h, the fluorescence intensity of CV@CaP in the tumor site was 3.5-fold higher than the initial (**Fig. S11**). However, the signal at the tumor sites of the mice injected with CV increased less over time, suggesting that the surface modification increased chlorella uptake and accumulation at tumor sites (**Fig. 4A**). The *ex vivo* fluorescence images of tumors and major organs revealed a similar distribution that CV@CaP mostly accumulated in the tumor and liver, little in other organs (**Fig. 4B**). Moreover, CV@CaP showed a higher tumor uptake at 4 h post-injection and was still detectable 24 h later, while its accumulation in other organs declined rapidly over time (**Fig. 4B, C**). Immunohistologic analysis of tumor sections showed that CV@CaP could accumulate in tumor tissues through tumor vasculature after intravenous injection. As shown in **Fig. S12**, CV@CaP (green arrows) colocalized with tumor vessels (red arrows) and accumulated in tumor tissues. The SEM images appeared to be another evident, a large number of CV@CaP were found in the tumor tissue after i.v. injection at 4 h (**Fig. 4D**). To conclude, the modified photosynthetic microalgae could be successfully accumulated in the tumor site and noninvasively tracked through fluorescence imaging.

The pharmacokinetic profile of CV@CaP was measured by fluorescence spectrophotometer to evaluate their time-dependent blood concentrations. As shown in **Fig. S13**, the blood circulation of the CV@CaP well conformed to the two-compartment model. After the first distribution phase (rapid decline, half-life: 0.84 ± 0.23 h), the CV@CaP had a long second elimination phase in circulating blood with a half-life of 13.42 ± 0.19 h, which was considered as the major process for CV@CaP clearance. Since the CV@CaP could be degraded in a simulated physiological environment, it might also be metabolized and thus be excreted from the body. We further investigated the clearance of CV@CaP and the results indicated that CV@CaP could undergo both renal and hepatobiliary clearance routes after intravenous injection, which were correlated with the high uptake of CV@CaP in liver, spleen and kidney (**Fig. S14**).

### Photoacoustic imaging-based oxygen saturation assessment

**Figure 5:**
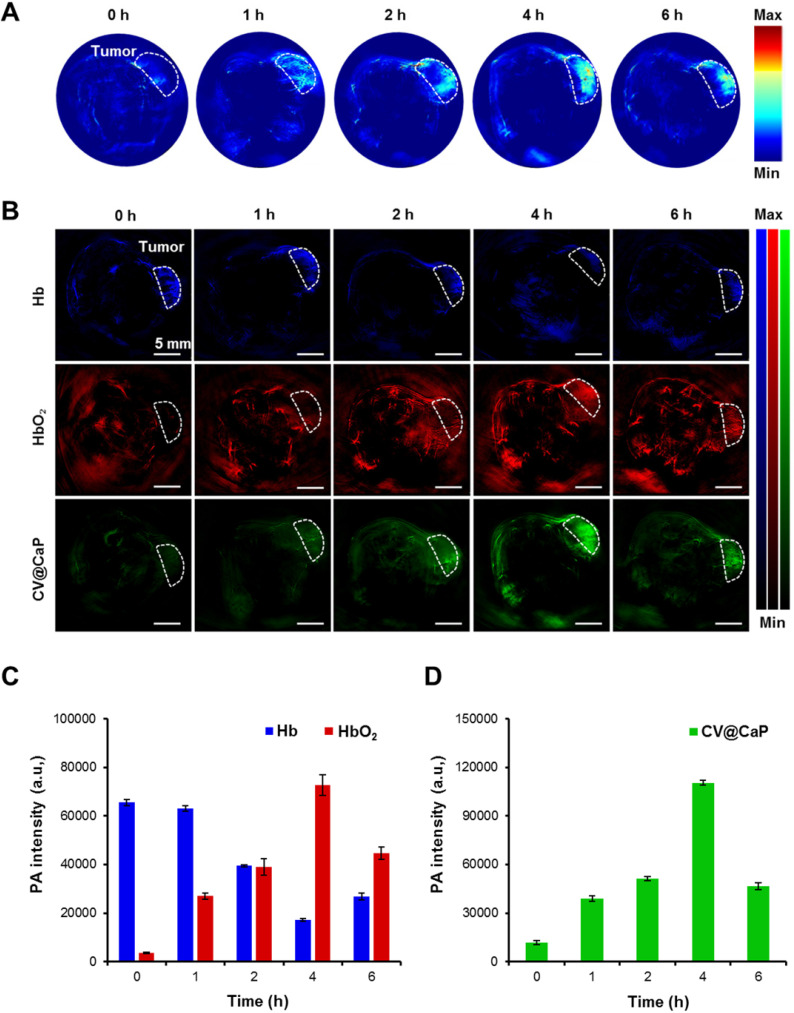
***In vivo* photoacoustic imaging and tumor oxygenation of CV@CaP.** (A) Time-dependent *in vivo* PA images of 4T1 tumor-bearing mice (white dashed circles: the tumor sites) after intravenous injection CV@CaP. (B) PA images of Hb (750 nm), HbO_2_ (850 nm) and CV@CaP (680 nm) of 4T1 tumor-bearing mice (white dashed circles: the tumor sites), indicating that CV@CaP effectively relieved tumor hypoxia by photosynthesis. Scale Bar = 5 mm. (C) Quantitative analysis of the PA intensity of Hb and HbO_2_ at the tumor sites. (D) Quantitative analysis of the PA intensity of CV@CaP at the tumor sites.

Owing to the abundant chlorophyll, CV@CaP exhibited a high absorption peak at 680 nm, which should generate an obvious PA signal at 680 nm. Hence, we next used a subcutaneous 4Τ1 tumor-bearing mice model to investigate the feasibility of applying CV@CaP to *in vivo* tumor photoacoustic imaging. As illustrated in **Fig. 5A**, the PA signals in the tumor site (white dashed circles) were started at 1 h, reached a peak at 4 h, and waned off at 6 h post-injection. The changes in PA intensity could be attributed to the accumulation of CV@CaP, consistent with the observations in fluorescence imaging. PA imaging also could be used to monitor the oxygen saturation by separately measuring the tissue concentration of deoxyhemoglobin (Hb) and hemoglobin (HbO_2_) at 750 and 850 nm, respectively 38, 39. The dynamic oxygenation process on modulating hypoxic microenvironment could be self-monitored via the PA response of CV@CaP, Hb and HbO_2_ in the tumor region (**Fig. 5B**). The PA signals of HbO_2_ (red) in the tumor site dramatically increased over time after CV@CaP administration, reaching the maximum until 4 h, while a reverse trend was found in Hb (blue), suggesting the optimized time window for radiation therapy. Importantly, CV@CaP photoacoustic images were basically co-located with HbO_2_ images, indicating the elevated oxygen level by CV@CaP in the tumor. Semiquantitative measurement of the amount of HbO_2_, Hb and CV@CaP in tumors showed that the PA intensity of HbO_2_, Hb and CV@CaP significantly changed to 26.2%, 2025.3% and 933.7% after 4 h post-injection, respectively (**Fig. 5C, D**). The results demonstrated that the excellent imaging performance of CV@CaP not only monitored its biodistribution *in vivo* but also dynamically visualized the supplementary oxygen in tumor regions, thereby achieving an FL and PA dual-modal imaging-guided cancer therapy.

### Synergistic anticancer therapy

**Figure 6:**
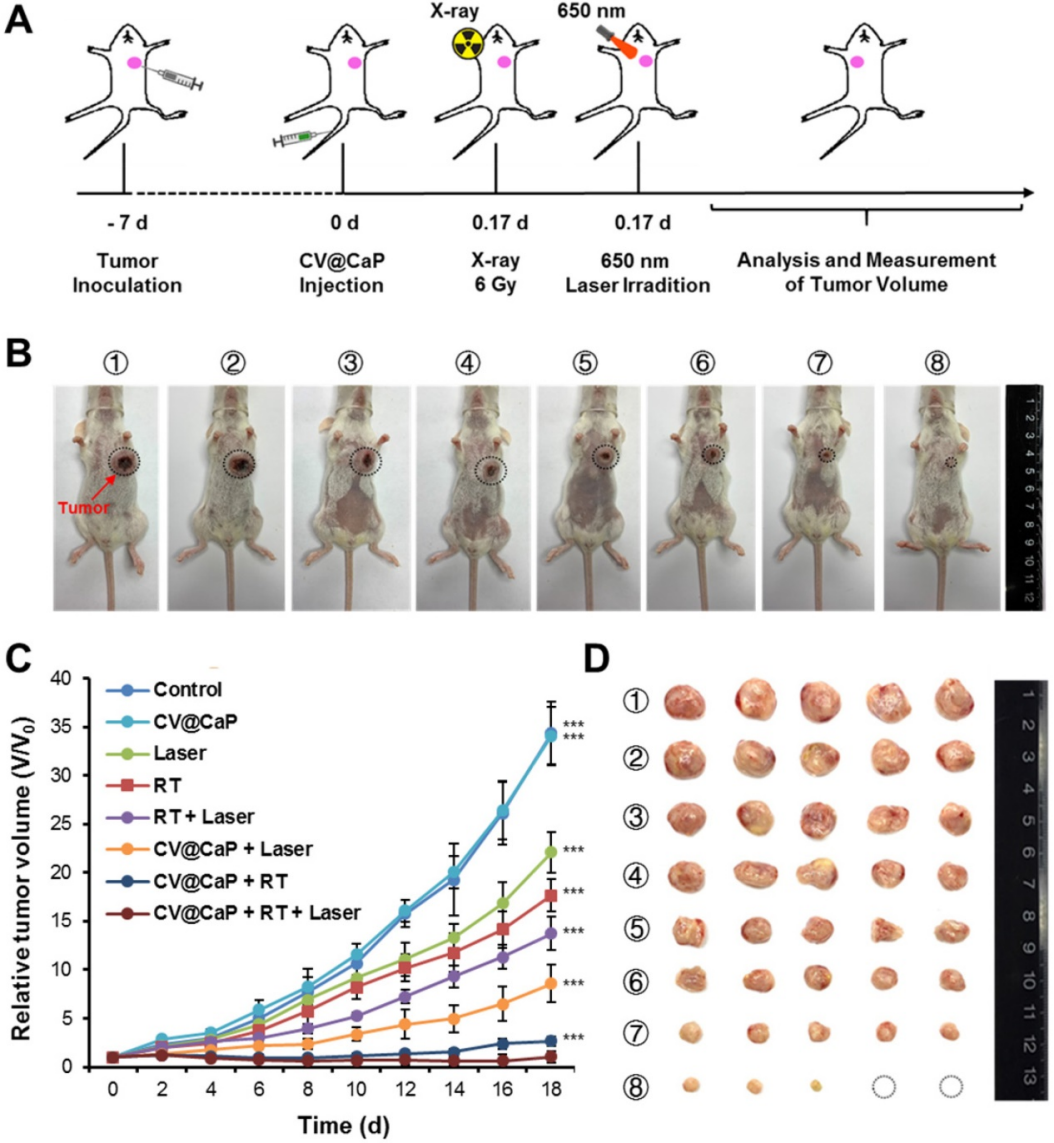
***In vivo* antitumor efficacy of CV@CaP in combination with radiotherapy and phototherapy.** (A) Experimental schematic of CV@CaP-mediated enhanced radio-phototherapy to inhibit tumor growth in the orthotopic 4T1 breast tumor model. (B) Representative photographs of the 4T1 tumor-bearing mice (black dashed circles: the tumor sites) at 18 days after different treatments (n =5). Treatments: 1) Control; 2) CV@CaP alone; 3) Laser alone (1.0 W/cm, 3 min); 4) RT alone (6 Gy); 5) RT + Laser; 6) CV@CaP + Laser; 7) CV@CaP + RT; and 8) CV@CaP + RT + Laser. (C) Tumor growth curves of mice in different groups (D) Photograph of the dissected tumors at 18 days in different groups. ***p < 0.001.

Next, the synergistic radiosensitizing and phototherapeutic effects of CV@CaP was evaluated *in vivo* using orthotopic 4T1 breast cancer mice model (**Fig. 6A**). The tumor sites of the 4Τ1 tumor-bearing mice in the synergetic group were subjected a 6 Gy dose of X-ray irradiation 4 h after i.v. injection, followed by exposure to a 650 nm laser (1 W/cm^2^) for 3 min. During the whole treatment, the tumor volume of 4Τ1 tumor-bearing mice was measured every other day. At 18 d, it was obvious that CV@CaP + RT + Laser group showed the most effective suppression on the tumor growth compared to other control groups (**Fig. 6B, C**). After 18 days, tumor tissues from each group were carefully harvested, weighed and photographed (**Fig. 6D** and **Fig. S15**). Likewise, CV@CaP + RT + Laser treatment achieved the highest inhibition rate (95.9%) as compared to Laser, RT and RT + Laser (IR = 24.7%, 41.3% and 54.2%, respectively). A similar trend was noticed in the spleen weights, with a 3.2-fold increase in the control group compared to the synergistic group, mainly due to granulocytic hyperplasia (**Fig. S16**) 40, 41.

### Immunohistochemical staining for therapeutic assessment

**Figure 7:**
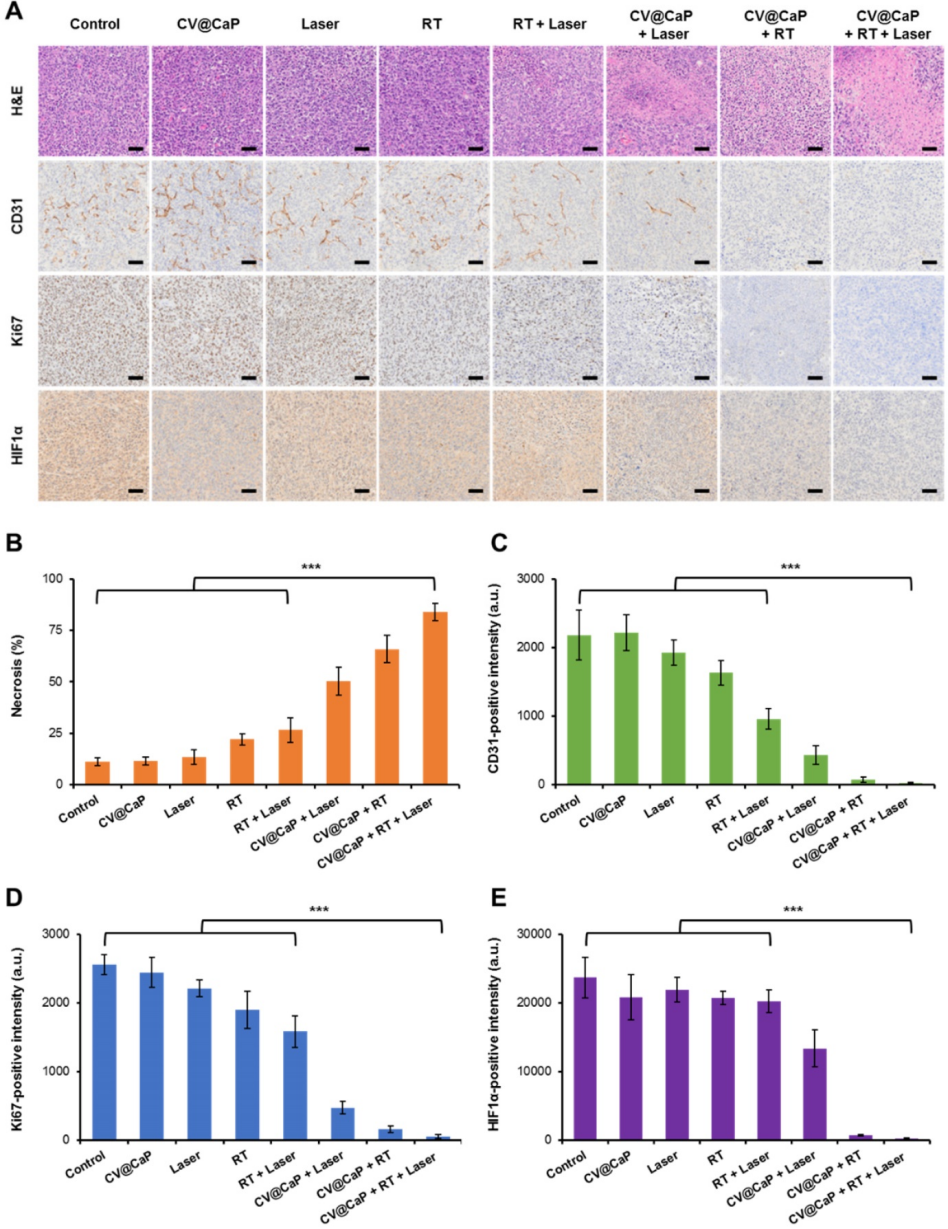
** Histological analysis of the tumor tissues.** (A) Representative H&E, CD31, Ki-67, and HIF-1α staining images of tumors (n = 5) at 18 days in different groups. Scale bar = 50 µm. Quantitative analysis of (B) necrosis, (C) CD31-, (D) Ki-67- and (E) HIF-1α-positive intensity at 16 days in different groups. ***p < 0.001.

The harvested tumor tissues were further analyzed using H&E staining and representative immunohistochemical analysis (CD-31, Ki-67, and HIF-1α) to assess the therapeutic efficacy of CV@CaP (**Fig. 7A**). H&E staining images showed different degrees of tumor cell necrosis in the treated groups, among which the necrosis rate of CV@CaP + RT + Laser group was 83.8% (**Fig. 7B**), while no obvious necrosis was observed in the control groups. Meanwhile, the suppression of different treatments on tumor angiogenesis was further assessed using CD-31 staining. As expected, the most dramatic decrease in tumor vascularization was noticed in CV@CaP + RT + Laser treatment (**Fig. 7C**). An evidently inhibited proliferating activity, manifested by the down-regulation of Ki-67, was also noted with the synergistic treatment. As shown in **Fig. 7D**, the expression of Ki-67, was significantly lower (p<0.001) in the CV@CaP + RT + Laser treated tumors (2.2%) compared to control, while it was as high as 86.5%, 74.1% and 62.0% in the Laser, RT and RT + Laser groups, respectively. Importantly, CV@CaP + RT + Laser significantly decreased the percentage of HIF-1α (p<0.001) in tumor tissues, which could be due to the combination of hypoxia amelioration and tumor inhibition (**Fig. 7E**). In agreement with the aforementioned results, these data further demonstrated the *in vivo* anticancer efficacy of CV@CaP + RT + Laser, thereby validating the potential of CV@CaP in radio-phototherapy.

### Anti-metastasis efficacy of CV@CaP

**Figure 8:**
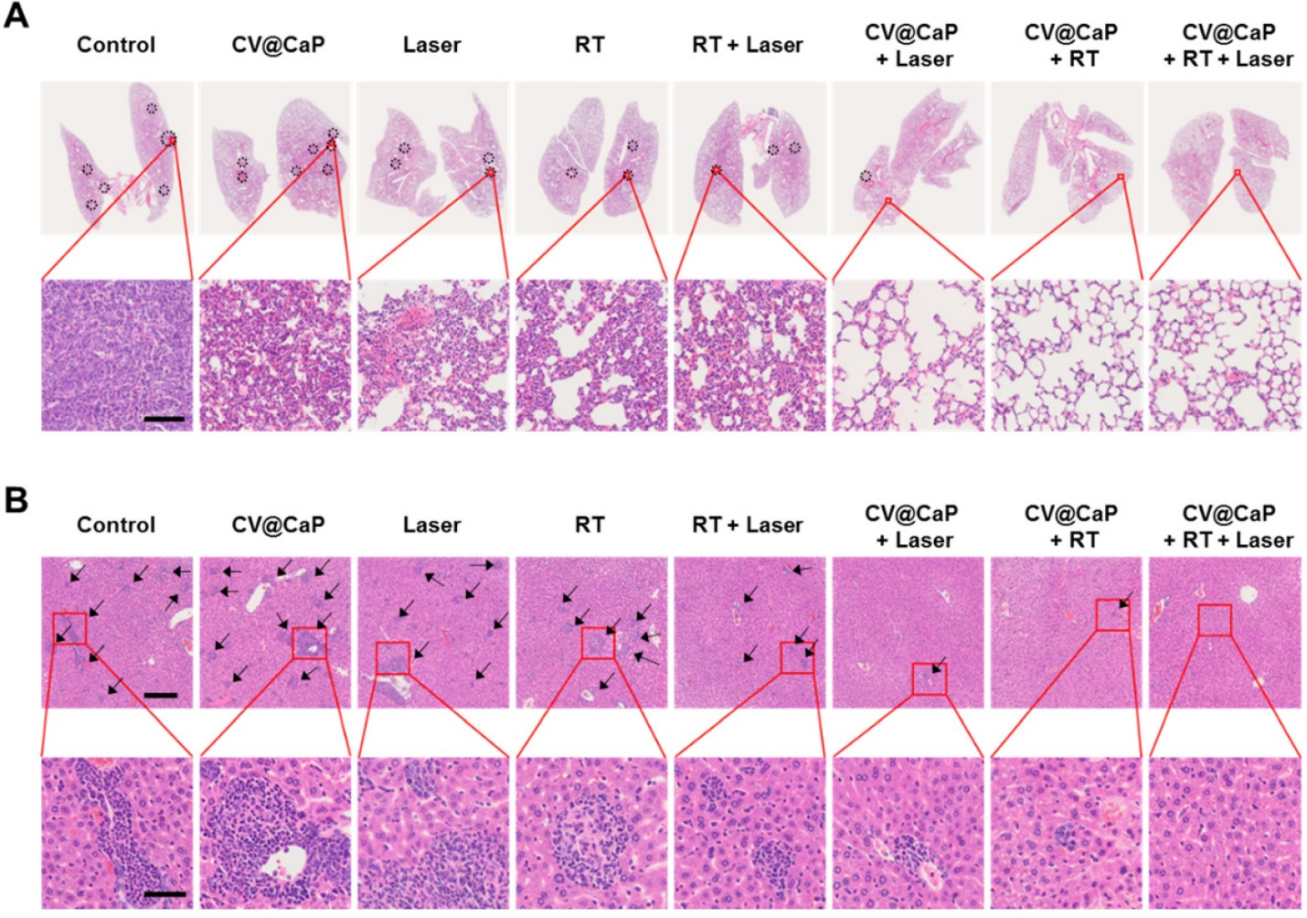
** The potency of CV@CaP combined radio-phototherapy in inhibiting distant metastasis of 4T1 cells.** (A) Representative photograph of lung pathological sections stained with H&E from each group of mice (n = 5) at 18 days (black dotted circles: metastatic foci). Scale bar = 200 µm. (B) Representative photograph of liver pathological sections (black arrows: micrometastases) stained with H&E; upper: scale bar = 200 µm; lower: scale bar = 50 µm.

Cancer metastasis is the leading cause for high mortality of breast cancer, such as lethal pulmonary and hepatic metastasis 42, 43. Inspired by the encouraging results in inhibiting the primary tumor growth, we further investigated the potential of CV@CaP-mediated radiosensitizing and phototherapeutice effects in anti-metastasis. As displayed in **Fig. 8A**, noticeable lung metastatic lesions could be detected in the control, CV@CaP, Laser, RT, and even the RT + Laser groups. In comparison, no visible metastatic foci were observed in the CV@CaP + Laser, CV@CaP + RT and CV@CaP + RT + Laser groups, likely owing to the capability of these treatments in preventing tumor spread. Likewise, a similar effect of suppressing liver metastasis was noticed in the CV@CaP-assisted treatments (**Fig. 8B** and **Fig. S17**). A large number of hepatic micro-metastases were also evidently found in the livers of the mice in the control, CV@CaP, Laser, RT, and RT + Laser treatments, whereas the micro-metastases were sharply reduced in the CV@CaP + RT + Laser-treated group.

Breast cancer stem cells (CSCs), are considered to be the main cause of disseminated tumor metastases, due to their resistance to therapy 44, 45. Therefore, we carried out tumorsphere assay to determine whether CV@CaP combined radio-phototherapy could modulate CSC populations in breast cancer. CV@CaP + RT + Laser treatment showed the most significant decrease in tumorsphere formation, and was significantly different from the other groups (**Fig. S18**). Taken together, these results indicated that CV@CaP + RT + Laser was not only effective in inhibiting primary tumor growth but also in suppressing distant metastasis of tumor by the highly efficient CSC killing.

### Pilot toxicity study

As a most important precondition, the biocompatibility of agents greatly affects its following clinical translation. Therefore, we also investigated the preliminary toxicity of CV@CaP. During the whole treatment, the body weight of 4Τ1 tumor-bearing mice was also measured every other day. Among all treated groups, no obvious difference in weight (**Fig. S19**) and any noticeable organ damage (**Fig. S20**) were observed, indicating the biosafety of the combinatorial treatment and CV@CaP agent *in vivo*. Furthermore, the long-term toxicity of CV@CaP was then evaluated by blood routine and blood biochemistry analysis of the mice i.v. injected with CV@CaP. Compared with the control group, no significant changes in blood routine and blood biochemistry parameters were observed 30 days after CV@CaP injection (**Fig. S21**), demonstrating the excellent biocompatibility of CV@CaP.

## Conclusion

In summary, we presented a living photosynthetic biosystem, mineralized photosynthetic microalgae (CV@CaP), for *in-situ* oxygen generation to alleviate tumor hypoxia, inhibit tumor growth and metastasis by the cascade combination therapy of oxygen-boosted RT and laser-induced phototherapy. We modified the photosynthetic microalgae with an artificial mineral shell to effectively deliver them to the tumor and perform the in-situ photosynthetic activity in the tumor, without causing apparent biological toxicity. The CV@CaP capable of photosynthesis relieved hypoxia both *in vitro* and *in vivo*, which effectively improved the radiosensitivity of the cancer cells, enabling an enhanced radiation treatment. Meanwhile, the chlorophyll released from CV@CaP, acted as a photosensitizer to generate cytotoxic ROS under 650 nm laser irradiation, in combination with thermal effects to kill tumor cells to achieve phototherapy. The cascade radio-phototherapy exhibited excellent capability both in terms of breast tumor growth and metastasis inhibition. We also found that the innate chlorophyll in CV@CaP could be used as both fluorescent and photoacoustic imaging agent, allowing non-invasively tracking *in vivo* and self-monitoring of the dynamic oxygenation process in tumor. Therefore, we believe this engineered photosynthetic microalgae-based biosystem would not only provide a novel strategy to overcome tumor hypoxia but also may contribute to alternative imaging-guided cancer treatment in the future.

## Supplementary Material

Supplementary figures and tables.
