# Supplementary material for: Calcium phosphate engineered photosynthetic microalgae to combat hypoxic-tumor by in-situ modulating hypoxia and cascade radio-phototherapy
Source: Theranostics. 2021 Jan 22;11(8):3580–94. doi: 10.7150/thno.55441 (PMC7914342; doi:10.7150/thno.55441)
Supplement: Supplementary file 1 — Supplementary figures and tables. [file thnov11p3580s1.pdf]

Supporting Information

Calcium Phosphate Engineered Photosynthetic Microalgae to Combat Hypoxic-Tumor by in-situ Modulating Hypoxia and Cascade Radio-Phototherapy

Danni Zhong[1,2], Wanlin Li[2], Shiyuan Hua[2], Yuchen Qi[2], Zhe Tang[1], and Min Zhou[1,2,3,#]

[1] Department of Surgery, the Fourth Affiliated Hospital, Zhejiang University School of Medicine, Yiwu 322000, China

[2] Institute of Translational Medicine, Zhejiang University, Hangzhou 310009, China

[3] State Key Laboratory of Modern Optical Instrumentations, Zhejiang University, Hangzhou, 310058, China

[#] **Address correspondence to**

zhoum@zju.edu.cn (Min Zhou);

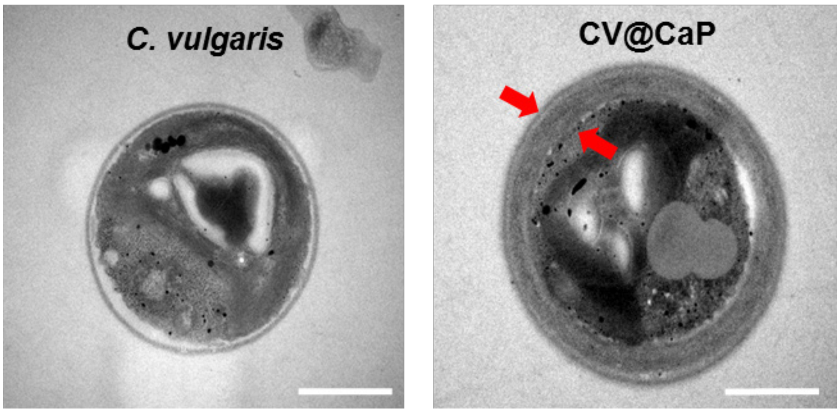

Fig. S1. TEM images of the bare *C. vulgaris* cell (left) and the mineralized CV@CaP cell (right), red arrows indicated the CaP shell. Scale bar = 1 μm.

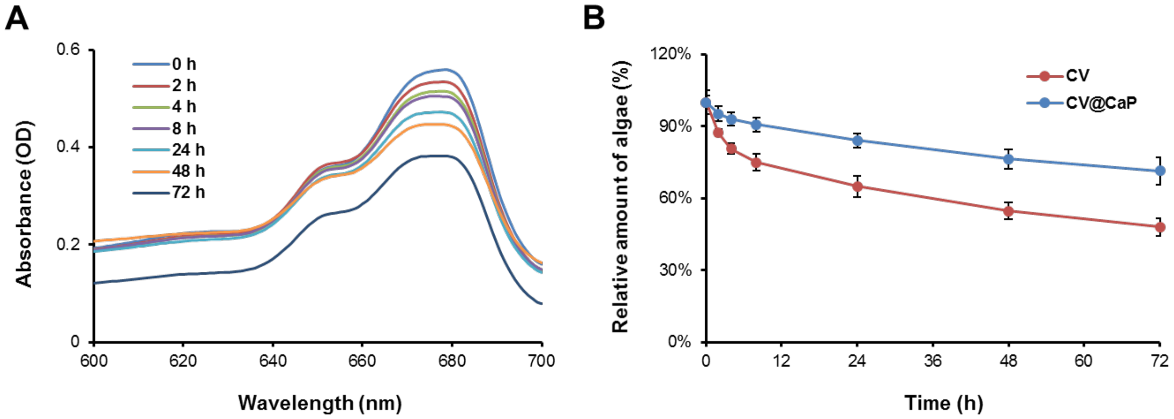

Fig. S2. Degradation of CV@CaP in 37°C PBS solution. (A) UV-Vis spectra of CV@CaP before degradation and after degradation of 2 h, 4 h, 8 h, 24 h, 48 h and 72 h, respectively. (B) Relative amount of CV and CV@CaP against degradation time.

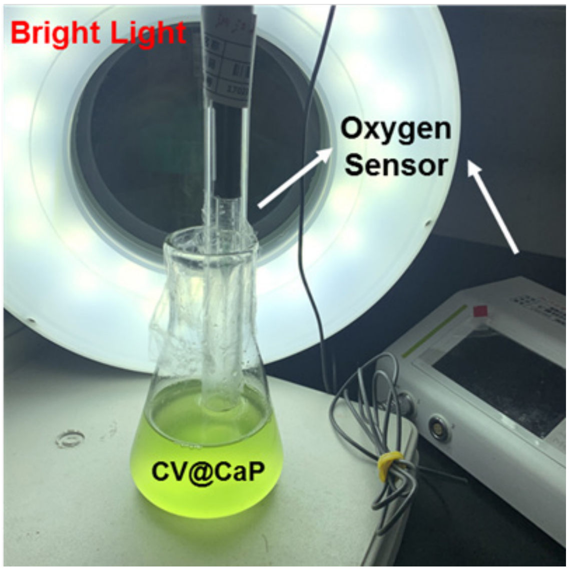

Fig. S3. Photograph of the oxygen sensor device testing the oxygen productions in the CV@CaP sample under the bright light.

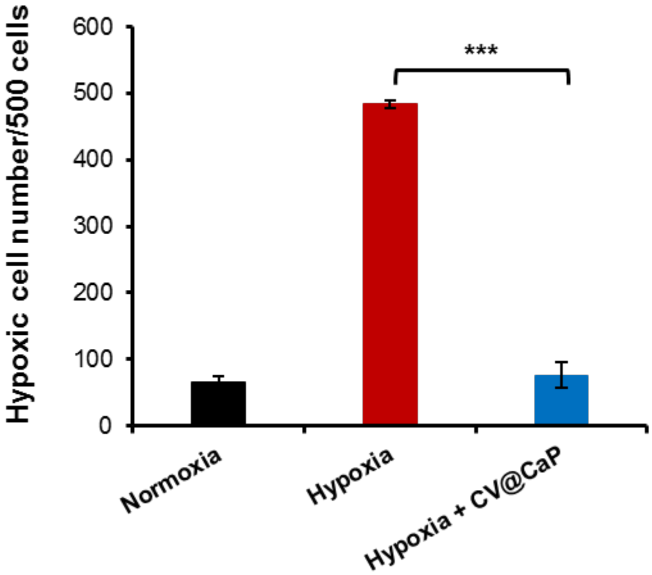

Fig. S4. Quantitative analysis of the hypoxic cell number after different treatments. ***$p < 0.001$.

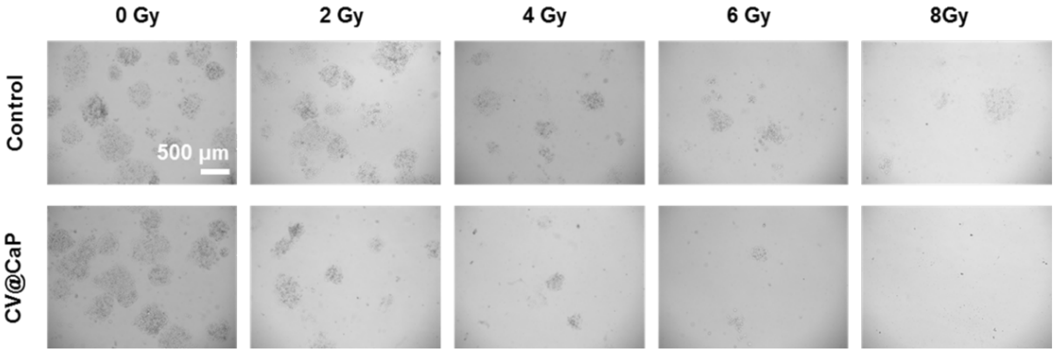

Fig. S5. Bright field images of the formation of 4T1 cell colonies after treated with or without CV@CaP under X-ray irradiation (0 to 8 Gy). Scale Bar = 500 μm.

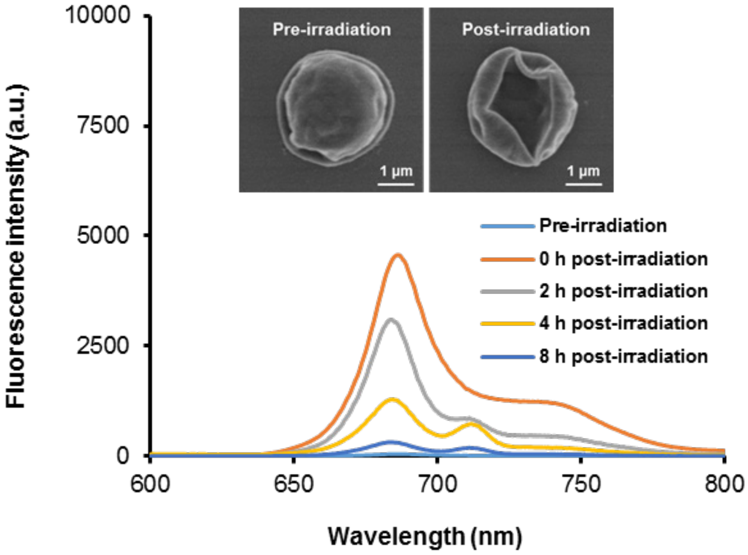

Fig. S6. Chlorophyll fluorescence intensity of the supernatant of CV@CaP collected pre- and post- X-ray irradiation (inset: SEM images of CV@CaP pre- and post-irradiation).

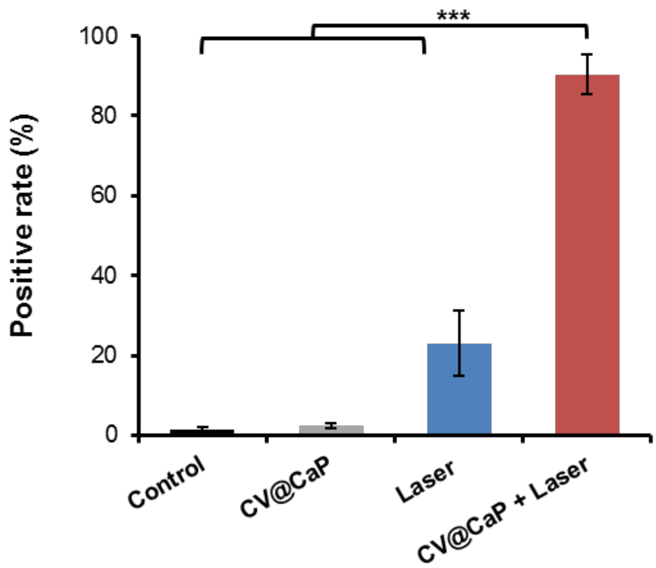

Fig. S7. Quantitative analysis of the ROS positive rate of each treatment in the fluorescence images. ***$p < 0.001$.

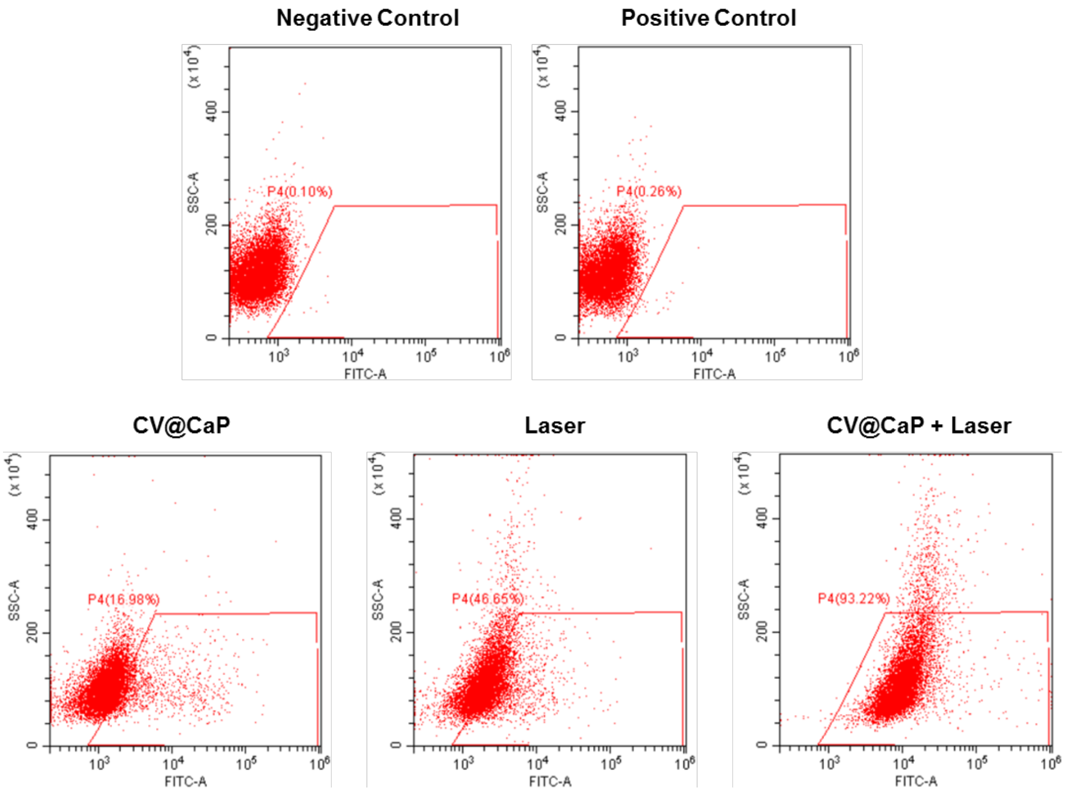

Fig. S8. Quantitative analysis of the ROS positive rate of each treatment in the flow cytometry analysis.

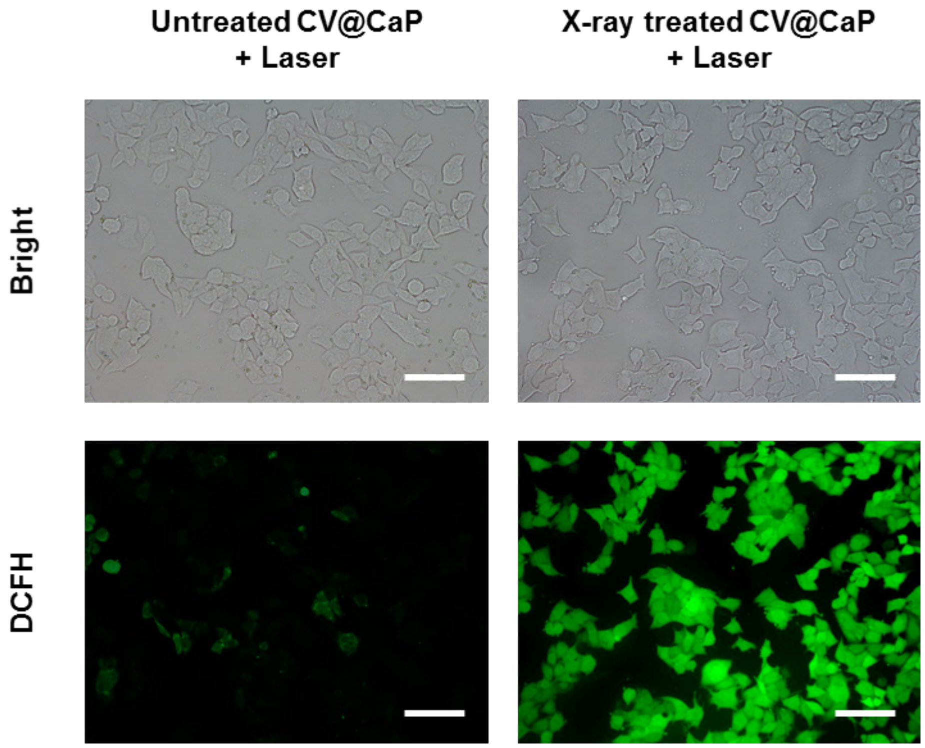

Fig. S9. Fluorescence images of 4T1 cells staining with DCFH-DA after administration with untreated CV@CaP + Laser or X-ray treated CV@CaP + Laser. Scale Bar = 100 μm.

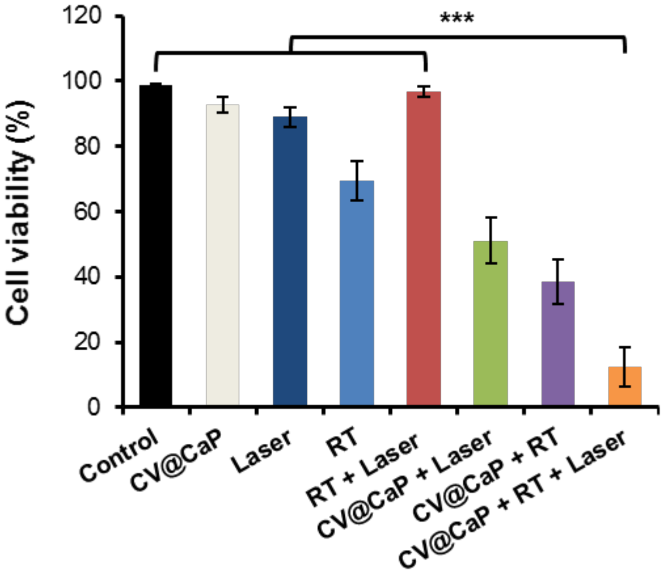

Fig. S10. Quantitative analysis of the cell viability of each group in live/dead staining assay. ***p < 0.001.

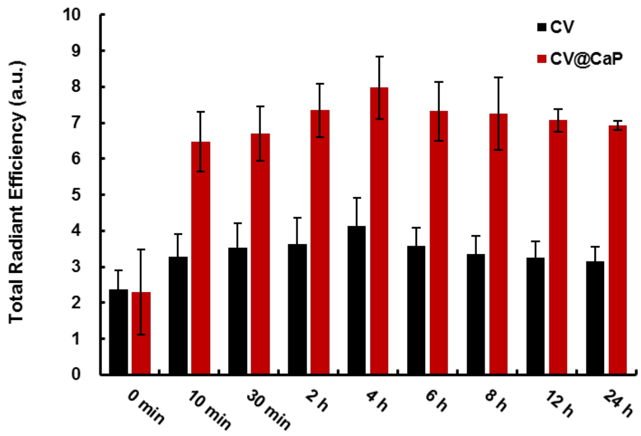

Fig. S11. Quantitative analysis of the total radiant efficiency at the tumor sites after intravenous injection of CV and CV@CaP.

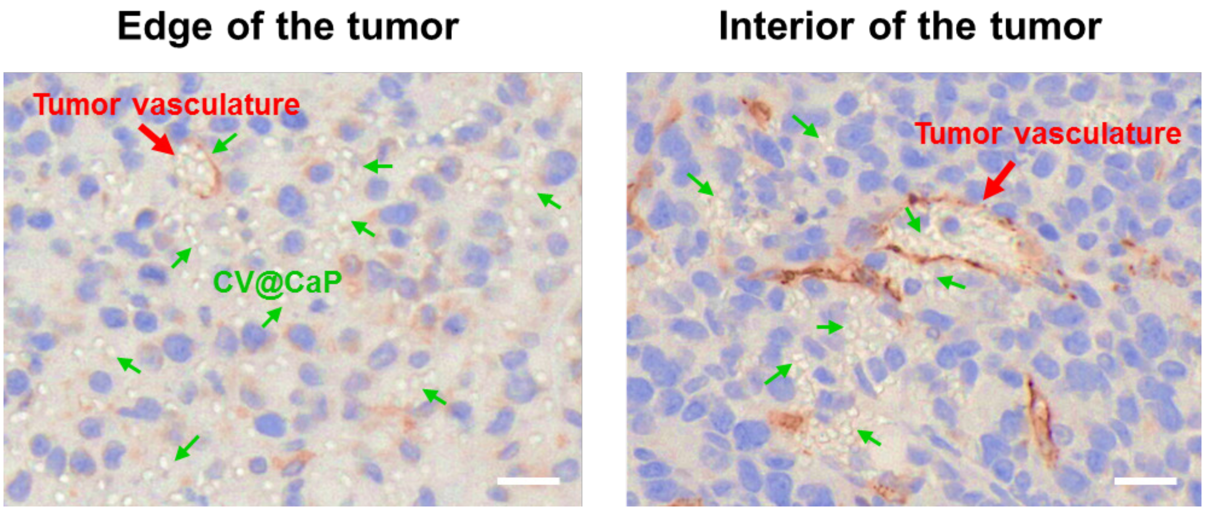

Fig. S12. CD31 staining of tumor tissue sections. Tumor vasculature was stained in brown, indicated by red arrows, and green arrows point to CV@CaP. Scale Bar = 20 µm.

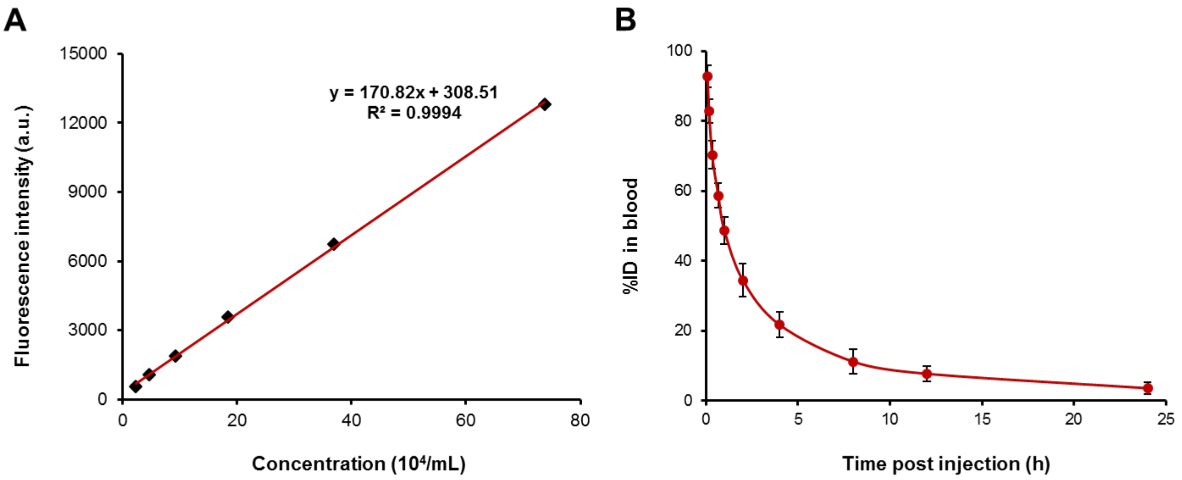

Fig. S13. (A) Fluorescence standard curve of CV@CaP in ethanol. (B) *In vivo* pharmacokinetic of CV@CaP in mice (n = 3) from 5 min to 24 h after intravenous administration. (Data is expressed as the percentage of the injected dose (% ID)).

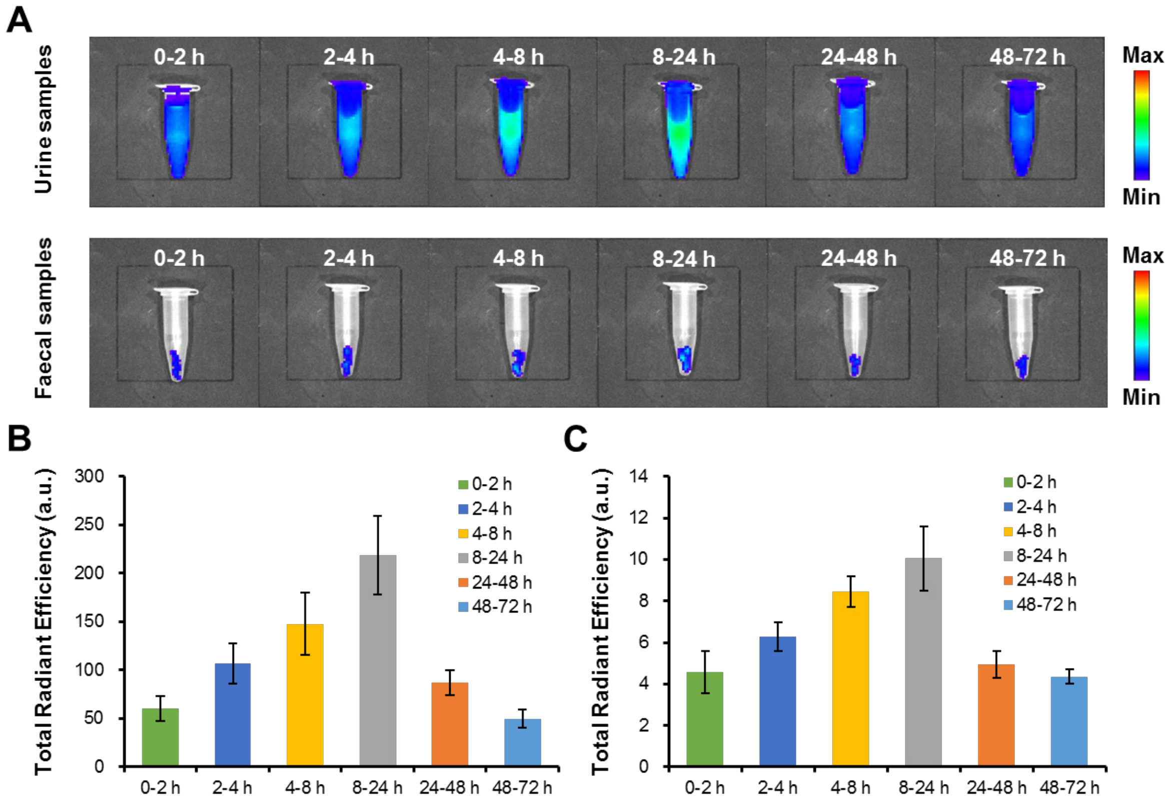

Fig. S14. Excretion analysis to evaluate renal and hepatic metabolism. (A) *Ex vivo* imaging of urine and faecal sample of mice (n = 3) collected at different times after i.v. injection of CV@CaP. (B) Quantitative analysis of the total radiant efficiency of different urine samples. (C) Quantitative analysis of the total radiant efficiency of different faecal samples.

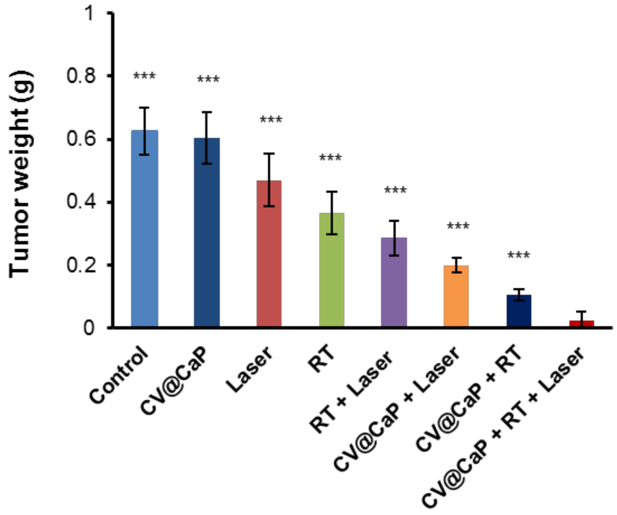

Fig. S15. Measurement of the tumor weight after various treatments.

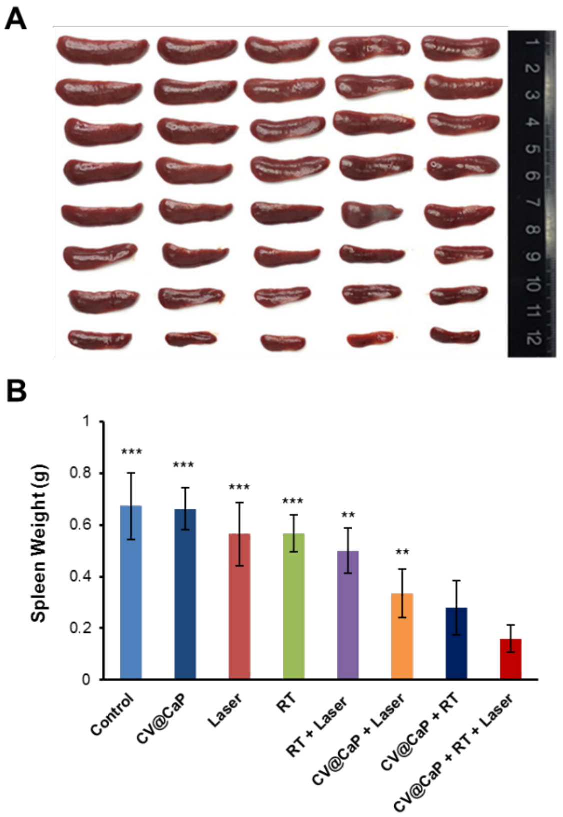

Fig. S16. (A) Photograph of the dissected spleens at 18 days in different groups. (B) Measurement of the spleen weight after various treatments. $^{**}p < 0.01$, $^{***}p < 0.001$.

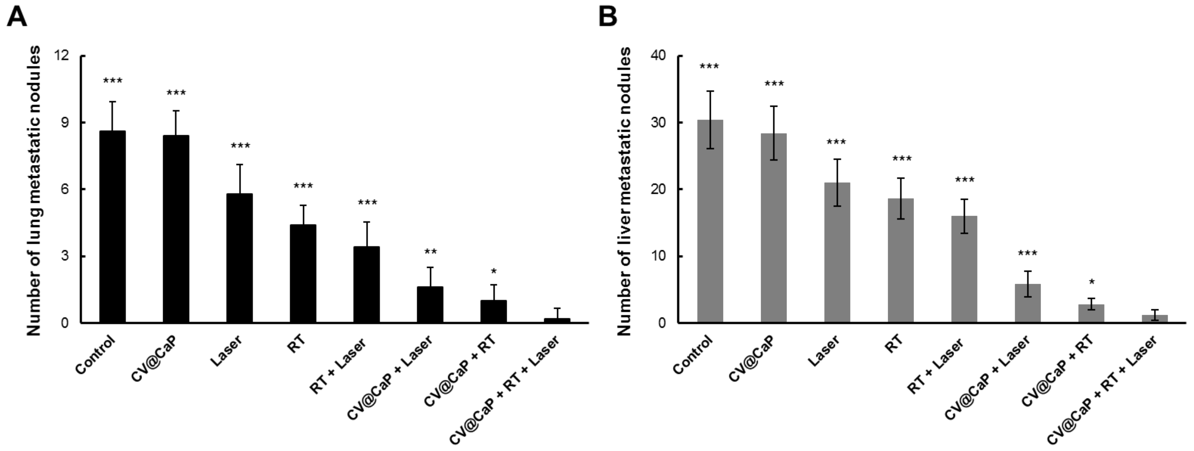

Fig. S17. The mean metastatic nodules of (A) lung and (B) liver tissues at 18 days in different groups. *$p < 0.05$, **$p < 0.01$, ***$p < 0.001$.

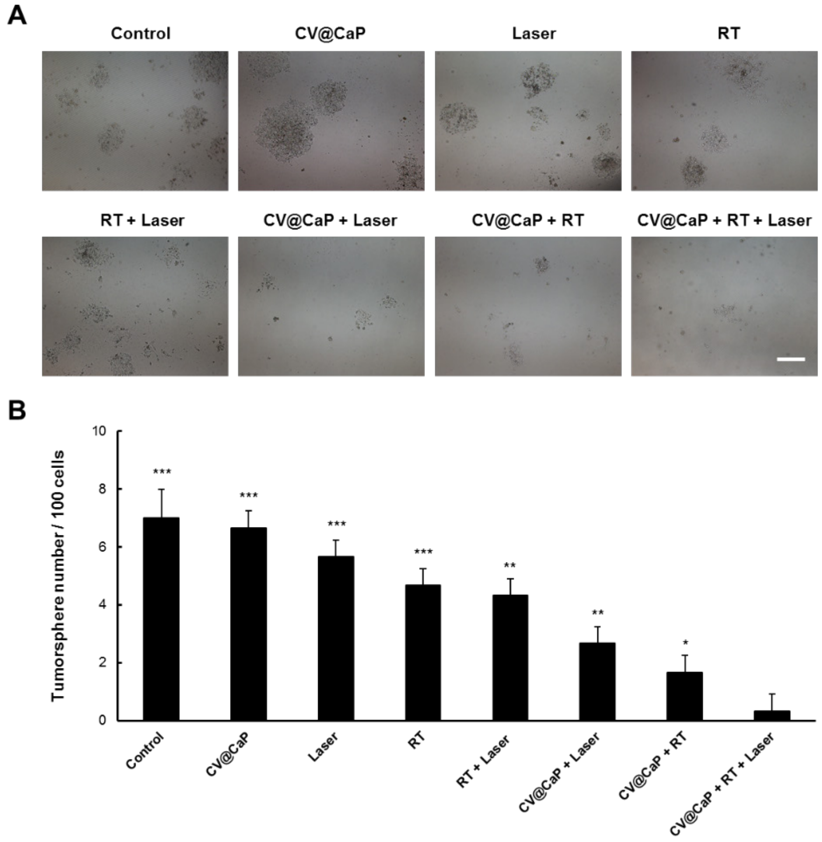

Fig. S18. (A) Representative images of 4T1 primary tumorsphere size 10 d after various treatments. (B) Quantitative analysis of the 4T1 primary tumorsphere formation 10 d after various treatments. *$p < 0.05$, **$p < 0.01$, ***$p < 0.001$.

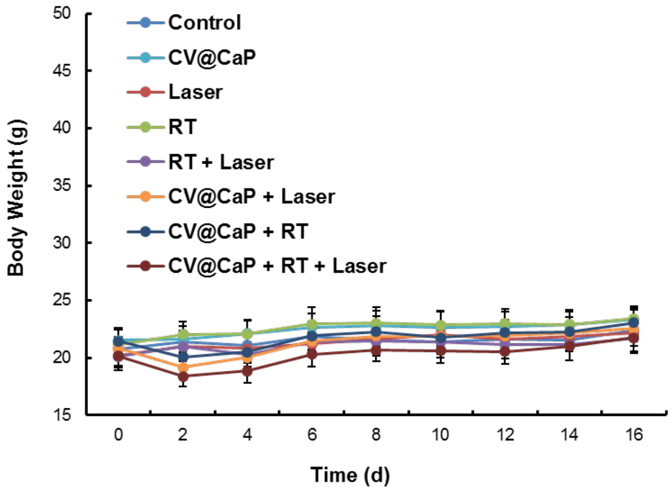

Fig. S19. Body weight of mice in different groups (n = 5). Treatments: Control; CV@CaP alone; Laser alone; RT alone; RT + Laser; CV@CaP + Laser; CV@CaP + RT; and CV@CaP + RT + Laser.

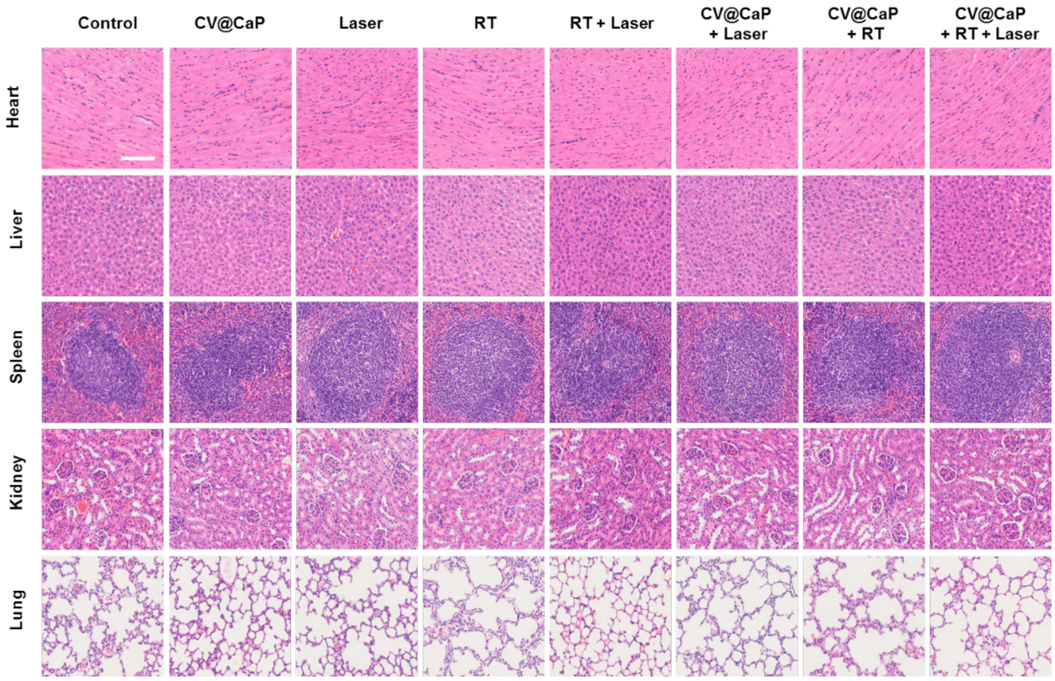

Fig. S20. H&E staining images of the major organs (Heart, Liver, Spleen, Kidney, and Lung) of the mice in each group (n = 5) at 18 days. Scale bar = 100 μm.

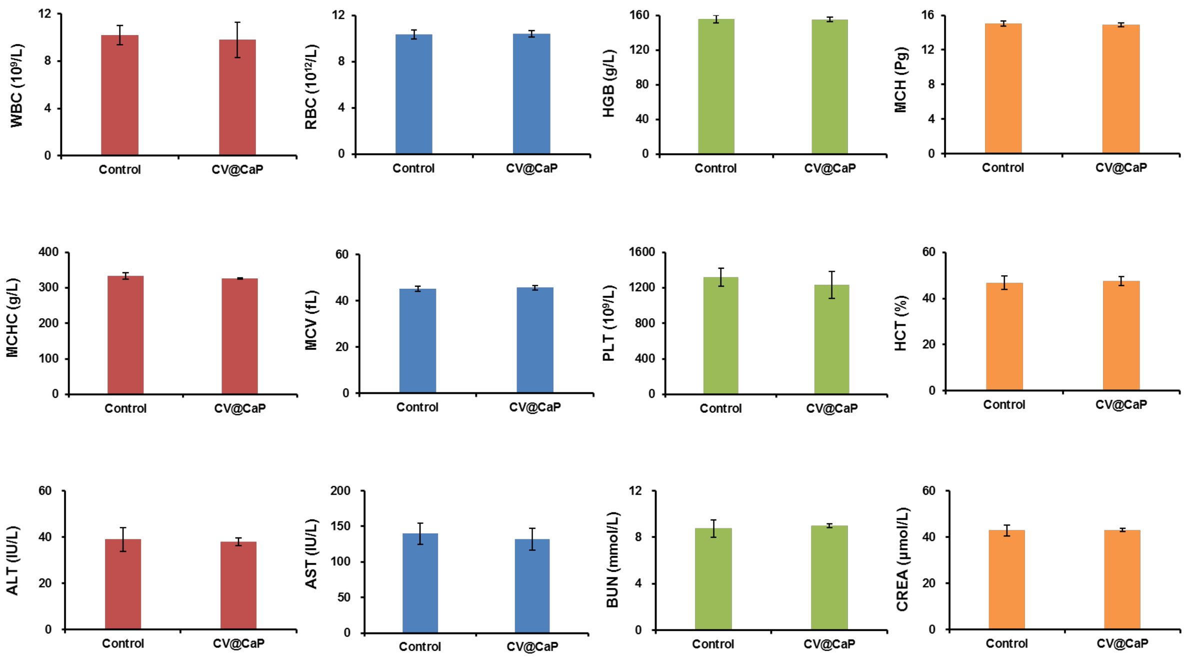

Fig. S21. Blood routine and blood biochemistry tests of the mice (n = 3) 30 d after i.v. injection of 200 μL of PBS or CV@CaP (1×10^7 cells/mL). WBC, white blood cells; RBC, red blood cells; HGB, hemoglobin; MCH, mean corpuscular hemoglobin; MCHC, mean corpuscular hemoglobin concentration; MCV, mean cell volume; PLT, blood platelet; HCT, hematocrit; ALT, alanine transferase; AST, aspartate transferase; BUN, blood urea nitrogen; CREA, creatinine.
